# Image Analysis Using the Fluorescence Imaging of Nuclear Staining (FINS) Algorithm

**DOI:** 10.1007/s10278-024-01097-8

**Published:** 2024-06-17

**Authors:** Laura R. Bramwell, Jack Spencer, Ryan Frankum, Emad Manni, Lorna W. Harries

**Affiliations:** 1 RNA-Mediated Mechanisms of Disease Group, Faculty of Life Sciences, Institute of Clinical and Biomedical Sciences, University of Exeter, Exeter, UK; 2 Translational Research Exchange @ Exeter, Living Systems Institute, University of Exeter, Exeter, UK

**Keywords:** Fluorescence imaging, Nuclear stain, Automated cell counting, Ki67, γH2AX

## Abstract

Finding appropriate image analysis techniques for a particular purpose can be difficult. In the context of the analysis of immunocytochemistry images, where the key information lies in the number of nuclei containing co-localised fluorescent signals from a marker of interest, researchers often opt to use manual counting techniques because of the paucity of available tools. Here, we present the development and validation of the Fluorescence Imaging of Nuclear Staining (FINS) algorithm for the quantification of fluorescent signals from immunocytochemically stained cells. The FINS algorithm is based on a variational segmentation of the nuclear stain channel and an iterative thresholding procedure to count co-localised fluorescent signals from nuclear proteins in other channels. We present experimental results comparing the FINS algorithm to the manual counts of seven researchers across a dataset of three human primary cell types which are immunocytochemically stained for a nuclear marker (DAPI), a biomarker of cellular proliferation (Ki67), and a biomarker of DNA damage (γH2AX). The quantitative performance of the algorithm is analysed in terms of consistency with the manual count data and acquisition time. The FINS algorithm produces data consistent with that achieved by manual counting but improves the process by reducing subjectivity and time. The algorithm is simple to use, based on software that is omnipresent in academia, and allows data review with its simple, intuitive user interface. We hope that, as the FINS tool is open-source and is custom-built for this specific application, it will streamline the analysis of immunocytochemical images.

**Graphical Abstract:**

In this paper, we describe a new tool—the Fluorescence Imaging of Nuclear Staining (FINS) algorithm. This tool can automatically count images of cells that are immunocytochemically stained with a nuclear protein of interest, producing a spreadsheet of counts and a user interface for review.

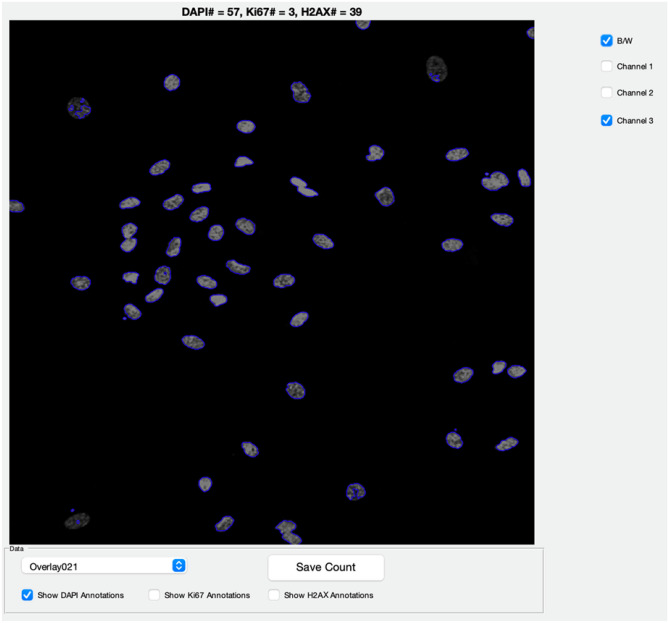

**Supplementary Information:**

The online version contains supplementary material available at 10.1007/s10278-024-01097-8.

## Introduction

There are many easy and open-source ways of analysing images of cell nuclei taken on a fluorescence microscope; however, there are far fewer options available for the analysis of immunocytochemically stained proteins that are co-localised specifically to the cell nucleus. Many software-based options are geared towards analysis of tissue sections which require slightly different considerations compared to cells grown on coverslips, as is the case here with our focus on immunocytochemically stained cells from tissue culture experiments. Manual counting is often used, but the method is subjective. Many researchers count cells in this type of image manually because it can be difficult for the typical academic laboratory researcher to find, build, or adapt a custom solution and/or justify the resources spent on creating and validating the process. Researchers may not be able to spend time learning how to use complicated open-source customisable image analysis pipelines or to justify the purchase of expensive proprietary software for a small job that is easy to achieve manually. There is a need for a simple, open-source technique to assess this type of image which will reduce inter-researcher subjectivity and analytical time.

An ideal solution would be specialised enough to be effective and enable some form of data review to enable checking of any anomalous data. Here, we describe a new image analysis algorithm—the Fluorescence Imaging of Nuclear Staining (FINS) analysis algorithm. This tool is specialised to differentiate and count cell nuclei based on any co-localised fluorescent signal appearing within the boundary of the nucleus. It uses a variational segmentation method with thresholding to define a fitting term. This creates a reliable base for nuclear segmentation, mirroring the way a researcher would manually count an image.

We have validated the FINS algorithm for two nuclear proteins of interest: Ki67 and γH2AX. Ki67 is a marker of cell proliferation, and γH2AX is a histone protein that is phosphorylated in the presence of DNA damage (specifically indicating a DNA damage repair response) [1–3]. When these two proteins are immunocytochemically stained, they exhibit punctate staining, where multiple foci may be present in the same nucleus. In this context, scientists may want to assess the presence or absence of fluorescent signals in counterstained nuclei in a binary fashion to gain an understanding of the number of positively and negatively stained cells in a culture in the context of assessment of proliferation capacity, cellular senescence or DNA damage burden. Therefore, we aimed to use datasets of images of three different primary human cell types and compare the performance of the FINS algorithm against researcher manual counts in terms of accuracy, reproducibility, quantification and analytical time.

## Methods

### Datasets

We access images for this study from several populations of cells. Set A (images 1–10) are images of human primary aortic endothelial cells (HAoEC). Set B (images 11–20) are images of human primary dermal fibroblasts (HDF). Set C (images 21–30) are human primary chondrocytes (HCH).

### Tissue Culture

HAoEC were cultured in Promocell Endothelial MV2 medium (C-22221, Promocell) supplemented with 2% foetal bovine serum (FBS) (16140071, Gibco™). All human primary dermal fibroblasts were grown in animal component-free conditions in Dulbecco’s modified eagle medium (DMEM) 1 g/l glucose + phenol red (31885023, Gibco™), 10% human serum (H3667, Sigma Aldrich), and 1% 10,000 units/ml penicillin—10,000 µg/ml streptomycin (15140122, Gibco™). HCH were cultured in DMEM F12 (21331020, Gibco™), 1% non-essential amino acids (NEAA) (11140035, Gibco™), 10% FBS (10270106, Gibco™), and 1% penicillin–streptomycin (15,140,122, Gibco™). Cells were grown on 13-mm coverslips in 12-well plates at approximately 30,000 cells per well.

### Immunocytochemical Staining

Cells were fixed using 4% paraformaldehyde and stored in Dulbecco’s phosphate-buffered saline (DPBS, 14190136, Gibco™). Cells were washed in DPBS and blocked using ADST [antibody diluent solution—triton: DPBS, 0.1 M L-Lysine (303341000, Thermo Scientific™), 1% w/v albumin (either human serum albumin fraction V (12668-10GM, Sigma-Aldrich) or bovine serum albumin, fraction V, fatty acid-free (10775835001, Roche)), Triton X-100 (A16046.AP, Thermo Scientific Alfa Aesar)] and 5% serum (either human serum (H3667, Sigma Aldrich) or FBS (16140071, Gibco™)) for 30 min. Cells were washed and primary antibodies at 2.5 µg/ml (suspended in ADST with 2% human serum or FBS) were applied overnight. After washing, a solution of secondary antibodies at 5 µg/ml and 4′,6-diamidino-2-phenylindole (DAPI, D1306, Invitrogen™) at 1 µg/ml was applied for 1 h, before mounting coverslips using Dako mounting medium (S302380-2, Agilent).

**Fig. 1:**
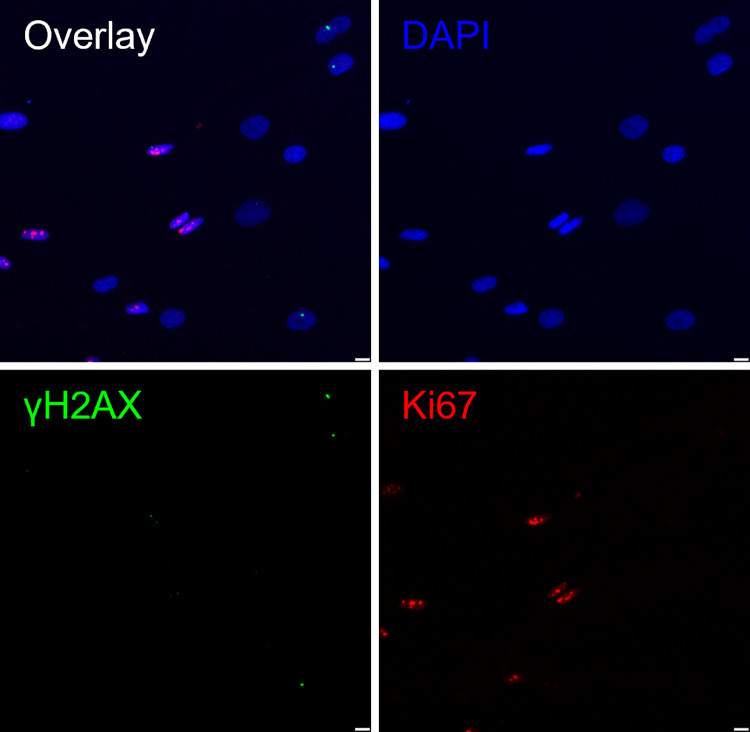
An example of an input image from set B. Note that the image is a cropped section and is in false colour to aid visualisation. The four panels show the overlay and the different channels: (top left) Overlaid image, (top right) DAPI staining, (bottom left) γH2AX staining, and (bottom right) Ki67 staining. The scale bars denote 10 µm

Antibodies were sourced from Abcam: Rb anti-Ki67 (ab15580, ab16667), Ms anti-γH2AX (ab26350), Alexa Fluor R 555 Goat pAb to Rb (ab150078, ab150086), and Alexa Fluor R 488 Goat pAb to Ms (ab150117). Fluorescence at 405 nm, 488 nm, and 555 nm was captured for DAPI, γH2AX, and Ki67, respectively, using the Leica DM4 B Upright Microscope at 10 × magnification and its associated image-capture software—Leica Application Suite X (LASX) 2019 3.7.1.21655v software (Leica Microsystems, Wetzlar, Germany). In Fig. 1, we present an example of a dermal fibroblast image (from dataset B), with a false colour visualisation of the corresponding Ki67, γH2AX, and DAPI channels (FINS denotes these as channels 1, 2, and 3, respectively). There is also an overlay image showing how the nuclear proteins (Ki67 and γH2AX channels) are co-localised with the nucleus (DAPI channel).

### Manual Counting Techniques

We have access to manual counting data for seven users. However, only five worked on sets A and C, with users 6 and 7 working on set B as well. As expected, there was a variety of methods used by the different researchers for counting. Five of the researchers did not export any images and stayed using the LASX software and its user interface to visualise the different channels and manually identify co-localised cells. Four of these researchers used the LASX software annotation tool to count and identify cells that had been counted, whilst the other kept mental track of both. One researcher exported images as.tiff files in false colour, opened them in basic image viewing software on a tablet computer, identified the cells with staining for a protein of interest by their false colour, and used a freehand drawing tool to check off cells after counting. They kept track mentally of the ongoing counts. One researcher exported images as.tiff files in false colour and opened them in ImageJ 1.47v software (US National Institute of Health, Bethesda, MD, USA) and then used the cell counter plugin to keep count of cells [4]. All researchers recorded their counts by typing them into a spreadsheet. When timing counts, a stopwatch was started when the image in question was open and ready for counting and stopped after the researcher had finished typing their counts for that image into the spreadsheet. All researchers were instructed to follow their normal procedure, whilst timing the duration from beginning to count the cells to inputting the data on a spreadsheet for set A. The counts were taken concurrently, but not necessarily continuously.

### FINS Algorithm

**Fig. 2:**
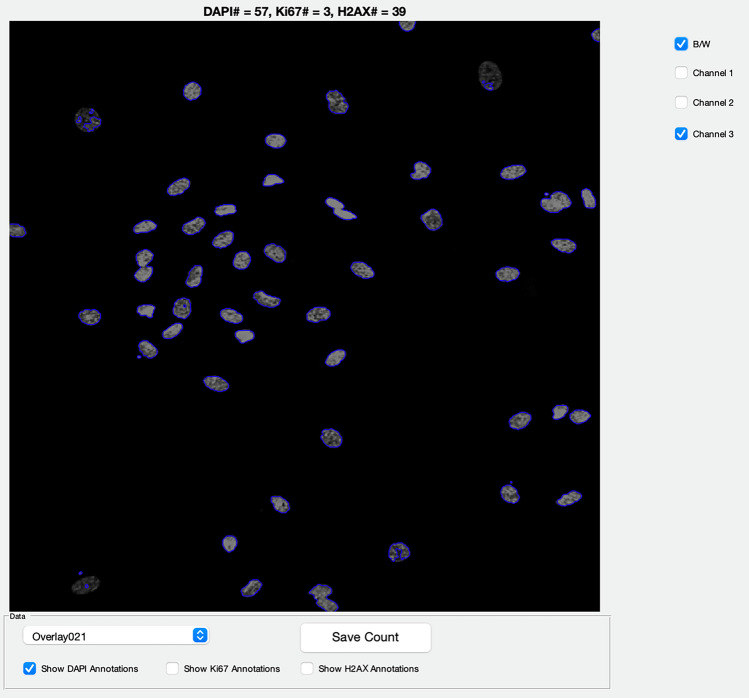
User interface of the FINS algorithm. The computed counts are displayed at the top of the image, and the user may alter the view to show different images in the dataset. The user can also alter the view to overlay different channels, to show the images in false colour or black and white, and to annotate how the algorithm has counted each channel

The FINS algorithm is a script created using Matlab software version R2017b. It is designed to quantify the number of nuclei in the DAPI channel and the number of nuclei containing any signal in the Ki67 and γH2AX channels. A researcher would download the folder containing FINS, move the associated.lif or.tiff files into the folder for counting, open the script in Matlab, and press “Save count”. The algorithm is left to run on multiple files placed in the folder but processes each image individually. After computation has finished, a simple, intuitive user interface (Fig. 2) enables the researcher to see how FINS has counted any image in the dataset. After reviewing the data if they wish, the researcher saves the count on the user interface which generates a timestamped output.csv file containing image names and all counts for each channel. Alternatively, the user may simply click to save the count and view all results in the.csv file without reviewing the image(s) on the user interface. The software cannot flag cells or images for review. Following review, a user may record adjusted counts and/or adjust thresholding parameters in the code to re-process the batch. Thresholding parameters are set to a default setting within the code, and apply to all images in a batch, but can be adjusted by the user. When taking the images on the microscope, it is essential to set the appropriate gain and exposure to ensure that the signal in the stain of interest is a “true” signal, but images can be adjusted with the microscope software and, if needed, exported prior to FINS analysis. Similarly, the code itself could be adapted and adjusted to user requirements.

## Results

### Development of the FINS Algorithm

The FINS algorithm works by initially computing the nuclear regions and then counting signals within this segment for the protein of interest. To compute the nuclear regions, we use a convex segmentation approach using a fitting term based on a thresholding of the image. It is designed to mimic the manual counting processes currently applied. We then iteratively search these regions in the Ki67 and γH2AX channels based on thresholds calculated from the image data. The thresholding parameters are set to a default setting within the code as calculated from our development data but can be adjusted by the user. In the following, we refer to the image data from the DAPI, Ki67, and γH2AX channels as ${z}_{\delta }(x)$, ${z}_{\chi }(x)$, and ${z}_{\gamma }(x)$, respectively, and we normalise them such that the intensities are scaled between 0 and 1. This allows for parameters to be defined in a consistent manner. The code is supplied in the Supplementary Information and can be adapted for additional uses.

### Nuclear Region Computation

When manually counting nuclei in the DAPI channel, the task is essentially a thresholding problem: count any segment where the fluorescence intensity is above a certain threshold. However, for the γH2AX and Ki67 counts, we also need to compute the boundaries of the counted DAPI regions. This presents two problems in the context of automating this procedure. The first is how to automatically select the threshold such that the algorithm can perform consistently across different cell types or multiple image types, etc. The second, more challenging issue, is how to account for noise in the image. The process that determines the boundaries of the nuclei in the DAPI channel is crucial as these regions are used to find proteins within the Ki67 and γH2AX channels.

We use a segmentation method, based on a variational approach, to partition the image into two regions: foreground (nuclei) and background. Generally, in the continuous setting, for an image $z\left(x\right)\in \left[\mathrm{0,1}\right]$ in the domain $\Omega \subset {R}^{2}$, the task is to compute disjoint subregions ${\Omega }_{1}$ and ${\Omega }_{2}$, such that ${\Omega }_{1}\cup {\Omega }_{2}=\Omega$, based on some partitioning criteria on the data $z\left(x\right)$ [5]. In this setting, we construct a data-fitting term using the optimal threshold ${t}_{\delta }$ based on Otsu thresholding [6]. To select this parameter, we divide the histogram of image intensities into two regions. This approach is an automatic clustering method which determines an optimal threshold value to minimise intra-class variance. This has been implemented efficiently in Matlab with the function “multithresh”. We use the convex relaxation approach of Chan et al. [7] and Goldstein and Osher [8], where binary labelling of the foreground and background is determined based on minimising the following energy functional:

1 $$\underset{u\in\;\{\mathrm{0,1}\}}{{\text{min}}}\left\{{\int }_{\Omega\;}g\left(x\right)\left|\nabla\;u\left(x\right)\right|\;dx+\;\lambda {\int }_{\Omega\;}f\left(x\right)u\left(x\right)\;dx\right\}$$

This involves total variation (TV) regularisation weighted by an edge function, $g(x)$, and some data fidelity term, $f(x)$. Equation (1) is a formulation that is designed to assign zeros (“background”) and ones (“foreground”) to each part of the image, such that the total value of the terms is minimised. The data term explicitly connects the functionality to the image data, and the TV term promotes smooth interfaces in the solution. Minimisation of this energy with respect to $u$ is a well-understood problem. One possibility is to relax the binary constraint such that intermediate values of $u(x)$ are permitted [7, 8]. Given that we are looking to find a regularised version of a thresholding procedure, we define an intensity fitting term in (1) as follows:

2 $${f\left(x\right)=f}_{\delta\;}(x)={t}_{\delta\;}-{z}_{\delta\;}(x)$$

where ${z}_{\delta }(x)$ is the image data in the DAPI channel. Edges are not particularly well-defined in this context, such that we can set $g\left(x\right)= 1$. According to these choices, the problem then consists of a two-phase variational segmentation problem that we consider in a conventional manner, relaxing the constraint on $u$, defined by the following:

3 $$\underset{u\in\;[\mathrm{0,1}]}{{\text{min}}}\left\{{\int\;}_{\Omega\;}\left|\nabla\;u\left(x\right)\right|\;dx+\;\lambda\;{\int }_{\Omega }{f}_{\delta\;}\left(x\right)u\left(x\right)\;dx\right\}$$

Here, the difference between (1) and (3) is that $g\left(x\right)$ and $f\left(x\right)$ have been defined, and the problem is now convex. This allows a global minimiser to be found. In this case, we use the Split Bregman approach to compute a minimiser [8, 9]. Many alternative methods are applicable in this case, such as the dual formulation or Chambolle and Pock’s algorithm [10–13]. We have found that the fastest method to obtain a solution is to define the initialisation, ${u}_{0}\left(x\right)$, as follows, as this is in close proximity to the global minimiser of (3) by definition.

4 $$u{}_{0}\left(x\right)=\left\{\begin{array}{c}1,\;{\text{for}}\;x\in\;{f}_{\delta\;}\left(x\right)<0\\\;0,\;{\text{for}}\;x\in\;{f}_{\delta\;}\left(x\right)\ge\;0\end{array}\right\}$$

However, we note that for fixed ${f}_{\delta }(x)$, the solution is independent of initialisation. We fix the value of the fitting parameter, $\lambda =20$, as we have found it to be appropriate for images of this type. We define the solution as ${u}^{*}(x)$. Based on the work of Chan et al. [7], Bresson et al. [11], and others, this will be approximately binary, such that any thresholding of the function will be a global minimiser of the original problem. We define the computed foreground region as follows:

5 $${\Omega\;}_{D}=\left\{x\in\;\Omega\;\right|\;{u}^{*}\left(x\right)>\beta\;\}$$

We select $\beta =0.5$ (although other values, such that $\beta \in (\mathrm{0,1})$, would yield a similar result). In the following, we use the binary form of the solution, ${u}^{*}$, denoted ${\Omega }_{D}$, to compute the counts of the nuclear proteins. The definition of (5) refers to the areas of the DAPI channel that are considered nuclei.

### Nuclear Protein Counts

**Fig. 3:**
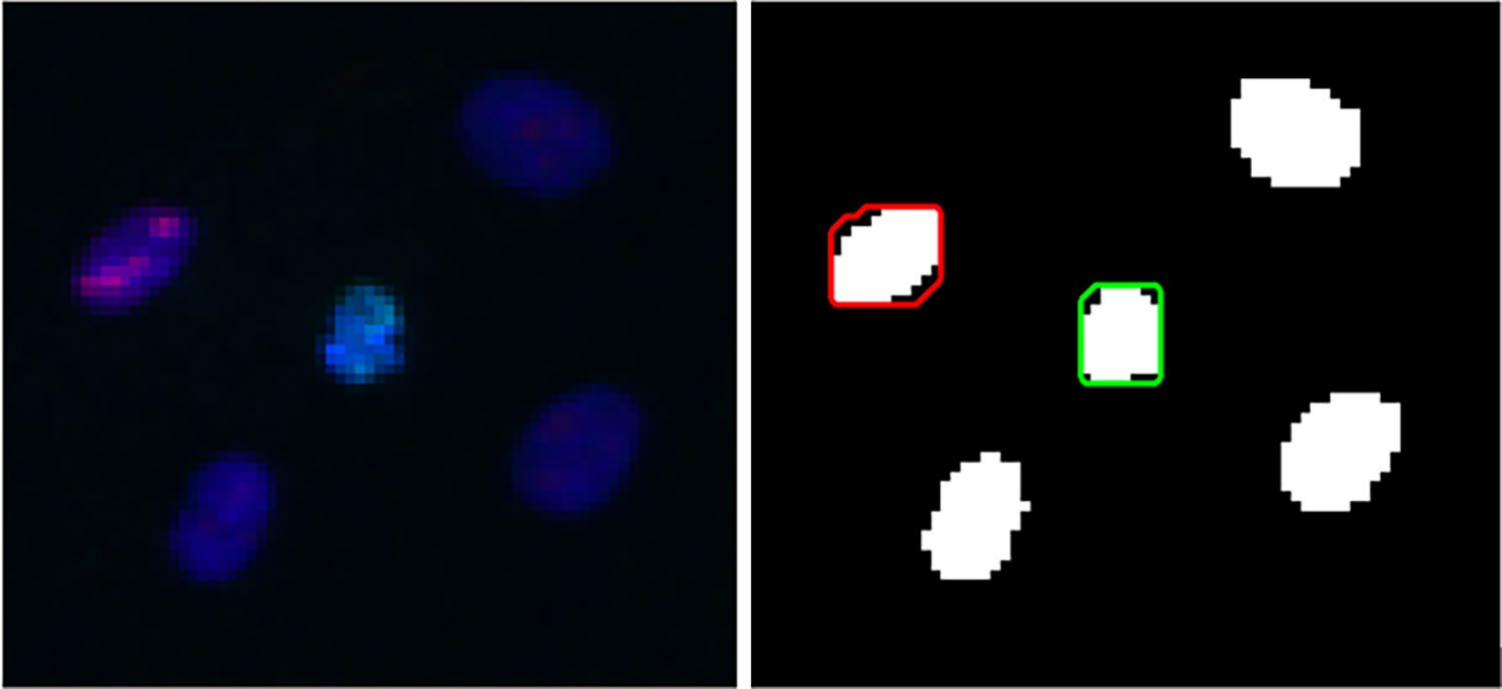
Example of the FINS processing. (left) cropped overlay image from set B. (right) corresponding FINS output. Binary regions represent ${\Omega }_{D}$ indicating five nuclei in this region. Red and green contours indicate Ki67 and γH2AX signals (respectively) which are contained within a nucleus

To simulate the manual counting procedure, we use the region calculated in the previous section, ${\Omega }_{D}$. This region will provide a space in which we can search for signals from nuclear proteins. However, unlike DAPI, we do not need to compute the regions of positive Ki67 or γH2AX signals. Instead, we only need to count the nuclei with the signal present. We treat the Ki67 and γH2AX channels in an identical way but describe the method here in general terms using Ki67 as our example. For this channel, we refer to the image data as ${z}_{\chi }(x)$. Using Otsu’s method, we determine a threshold ${t}_{\chi }$ on the entire image. For each disconnected region ${\Omega }_{D}^{i}\in {\Omega }_{D}$, we determine whether ${z}_{\chi }\left(x\right)>{t}_{\chi }$ for any $x\in {\Omega }_{D}^{i}$. If so, this nucleus is considered to be positive for Ki67. If not, then it is negative for Ki67 (illustrated in Fig. 3). This is calculated for $i = 1, \dots , n$, such that the maximum count for Ki67 is $n$ (i.e., the total number of nuclei calculated by the process of determining the nuclear regions in the DAPI channel). For cases where Otsu’s method does not provide an adequate threshold, we defined a floor on the parameter ${t}_{\chi }$ such that ${t}_{\chi }= {t}_{f}=0.1$ to enable the image to be processed. This process is repeated for the γH2AX channel (with ${z}_{\gamma }\left(x\right)$ and ${t}_{\gamma }$).

### Validation of the FINS Algorithm

In this section, we present results for the proposed algorithm, FINS, compared with the manual counts of researchers 1–7 (where available). We are interested in two aspects of the results of the proposed algorithm: the reliability of the counts for each channel in comparison with the manual data and the improvement in the time taken to compute a result. The results consist of three distinct datasets, which we call sets A, B, and C, respectively. Set A consists of images 1–10 (human primary endothelial cells; HAoECs), set B consists of images 11–20 (human primary dermal fibroblasts; HDFs), and set C consists of images 21–30 (human primary chondrocytes; HCHs). For each image, we have a manual count from the Ki67, γH2AX, and DAPI channels. Researchers 1–5 provided counts for all three sets, and researchers 6 and 7 provided additional counts for set B. We also have data on the time taken to count each image for set A from researchers 1–4. Counts from the FINS algorithm can be reviewed with the user interface, but for more rigorous comparisons, the counts computed by FINS are not reviewed or adjusted by any researcher.

### Count Comparisons

**Fig. 4:**
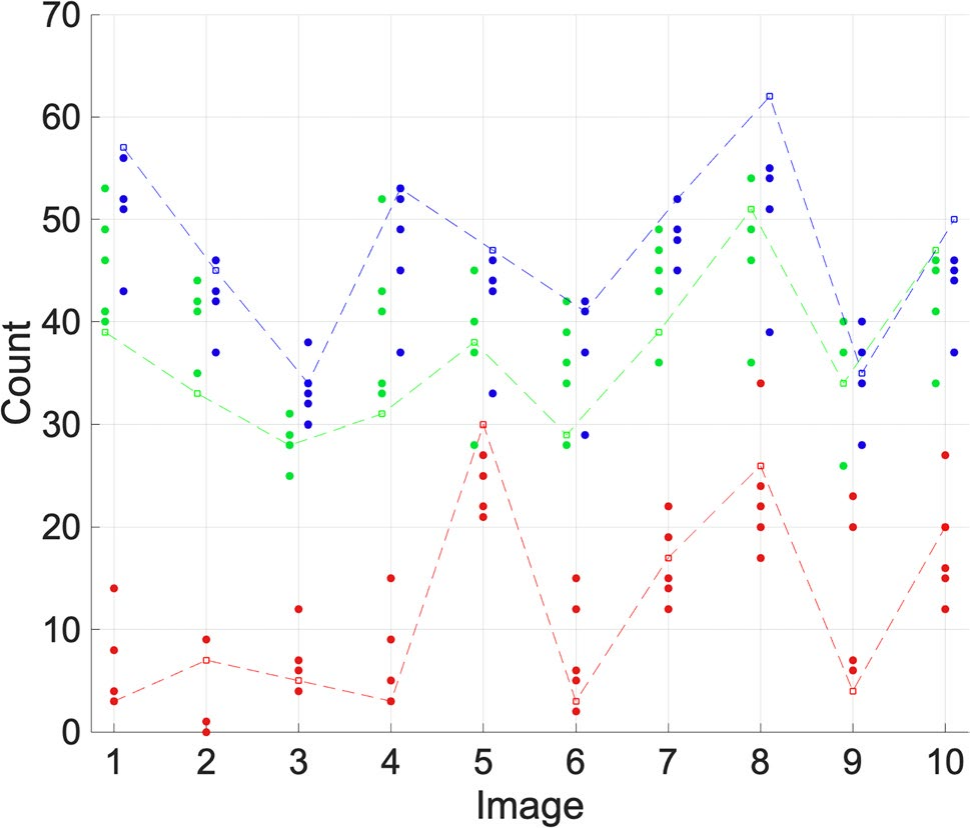
Ki67 (red), γH2AX (green), and DAPI (blue) count results for set A. Researcher counts are indicated by a filled circle. Algorithm counts are indicated by an empty circle and are joined by a dashed line for clarity

**Fig. 5:**
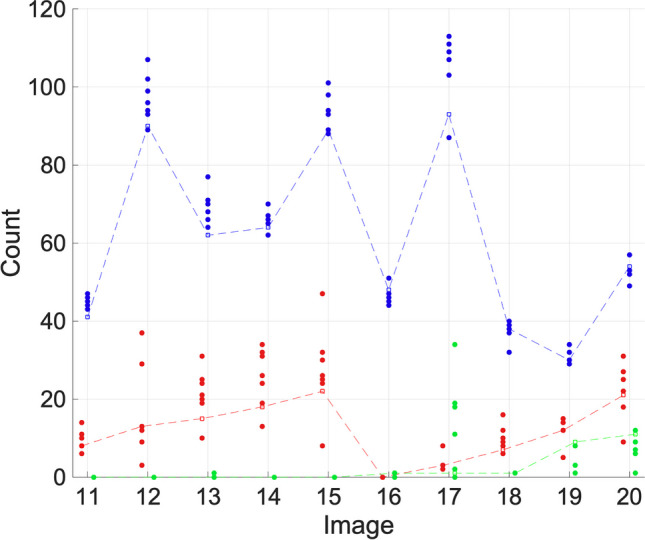
Ki67 (red), γH2AX (green), and DAPI (blue) count results for set B. Researcher counts are indicated by a filled circle. Algorithm counts are indicated by an empty circle and are joined by a dashed line for clarity

**Fig. 6:**
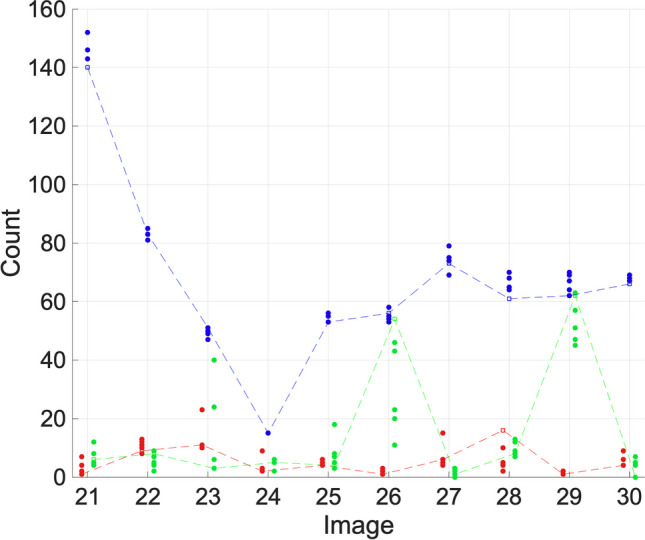
Ki67 (red), γH2AX (green), and DAPI (blue) count results for set C. Researcher counts are indicated by a filled circle. Algorithm counts are indicated by an empty circle and are joined by a dashed line for clarity

We present a visualisation of the results in Figs. 4, 5, and 6. Each figure displays every manual count for each dataset as well as the result achieved by the proposed algorithm. The results are split into Ki67, γH2AX, and DAPI counts. The count computed by the algorithm is connected by a dashed line to distinguish it from the rest of the data.

Figure 4 concerns set A. It should be noted that the FINS algorithm tends to be at the higher end of the range of the researchers for the DAPI channel, but this appears acceptable and mitigated for in context. For images 1, 5, 8, and 10, the FINS count is higher than the maximum of the researcher counts. For images 1 and 5, this is a small difference, with FINS being 1.79% and 2.17% over the maximum researcher count. However, for image 8 it is 12.7% over, and for image 10 it is 8.7%. For image 8, the researcher counts’ range is 39–55, with FINS computing 62. The algorithm is closer to the counts of researchers 1, 2, 3, and 5 (DAPI = 55, 51, 54, and 51, respectively) than researcher 2 is to the others (DAPI = 39). In this image, there are a lot of borderline cells meaning the researcher counting 39 nuclei is probably using a subjective method, perhaps omitting cells that are at the border of the image. In contrast, FINS is overcounting likely due to some detritus in this particular image. Nuclear size can often vary, meaning that to enable successful counts of these differently sized nuclei, there is an unavoidable risk of some cell detritus being counted. However, the FINS user interface allows for post-analysis inspection to mitigate this issue. For image 10, FINS is closer (DAPI = 50) to the counts of researchers 1, 2, 3, and 5 (DAPI = 46, 44, 45, and 46, respectively) than researcher 2 is (DAPI = 37). In context, this demonstrates that FINS is similar to the manual counts. Ki67 is within the range of the researchers’ counts for 80% of images. For γH2AX cells, three images are below the range of researcher counts: image 1 is 2.5% below, image 2 is 5.71%, and image 4 is 6.06% (image 10 is above the range by 2.17%). These are relatively small differences, but we should note that when combined with the minor over-counting for DAPI this could have implications for the conclusions from the data, i.e., the ratio of cells stained for γH2AX to DAPI may be lower than the true percentage due to the γH2AX undercount and DAPI overcount. If this is an unacceptable margin of error to a researcher, then it is easy to mitigate against by using the user interface’s review window.

Figure 5 shows the results for set B. FINS performs comparably with the manual counts. For Ki67, FINS is within the range of the researchers’ count for all images. For γH2AX, FINS is within the range for 90% of images. The minor trend of over-counting in the DAPI channel in set A does not seem to have been repeated here in set B as FINS is within the researcher count range for 80% of images and is under the mean count seven times and over three times. It should be noted that for this data set, we have an additional two users providing manual counts. The Ki67 and γH2AX numbers are very low for set B which creates a large potential for unreliability (even a small amount of over-counting would be very significant here), but FINS appears to be reliable when the number of cells in a channel is low. For the γH2AX data, there are four images (11, 12, 14, 15) where all users agree that there are no γH2AX stained-cells present, and FINS also computes a zero for these cases.

Set C, shown in Fig. 6, has consistent data. For Ki67 and γH2AX, the FINS results are within the researchers’ count range for 90% of images. As previously discussed, variation between images can arise due to many reasons, such as the image capture settings, so it may be that the minor over-counting trend in the DAPI channel in set A was a result of the nature of the images, as this trend is not observed in Set B or Set C. For DAPI, we observed no trend to over-count (FINS is under the mean count six times, over twice, equal twice), but only 70% are within the researchers’ range. However, it is clear that there is a broad consensus between the algorithm and the users (image 24 even has all agreeing precisely). Expressed as percentages of the mean, the researcher counts show a range of 6.09% for image 21, 9.04%, for image 28, and 2.96% for image 30. For these three cases, the FINS results are only slightly below the lowest count (3, 3, and 1, respectively); FINS computes a count which is 94.7% of the mean count for image 21, 91.9% for image 28, and 97.6% for image 30. On the whole, the algorithm is consistently accurate for each channel.

**Fig. 7:**
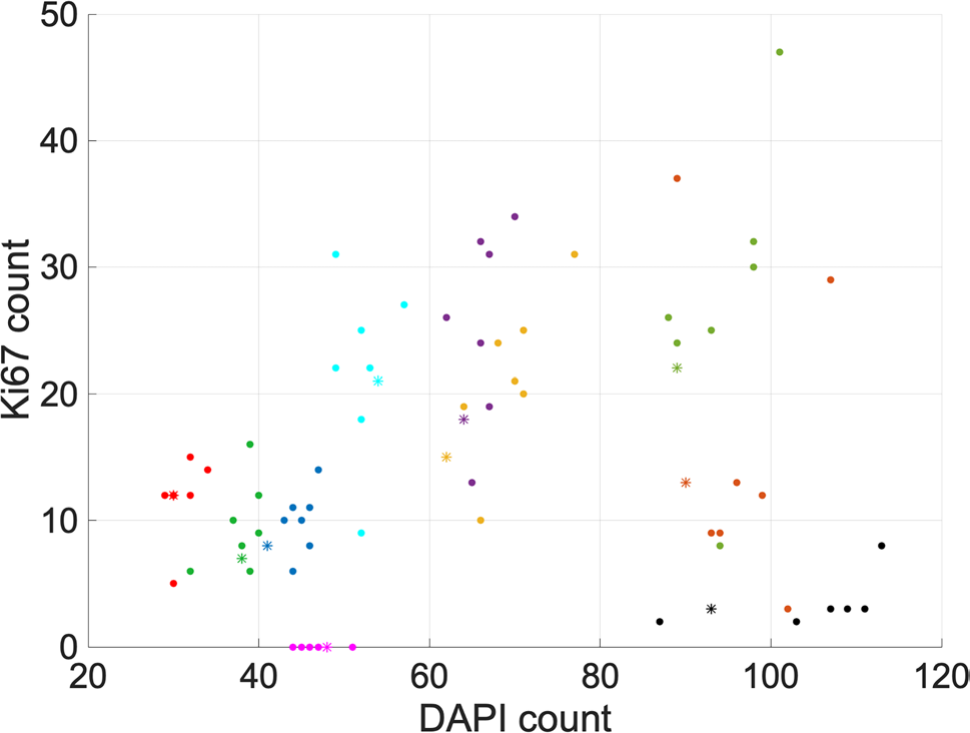
DAPI versus Ki67 counts for set B. FINS count is indicated by an asterisk “*”. Filled circles indicate researcher counts. Images 11–20 are indicated by dark blue, dark green, yellow, purple, light green, pink, light blue, orange, red, and black, respectively

**Fig. 8:**
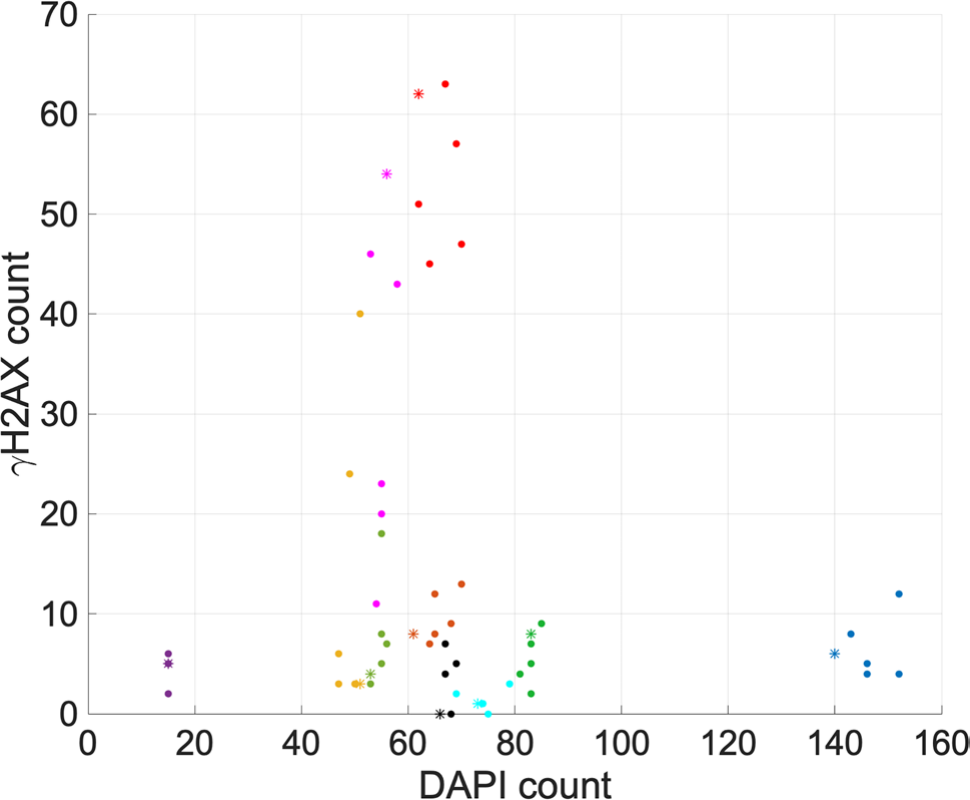
DAPI versus γH2AX counts for set C. FINS count is indicated by a *. Filled circles indicate researcher counts. Images 21-30 are indicated by dark blue, dark green, yellow, purple, light green, pink, light blue, orange, red and black respectively

**Fig. 9:**
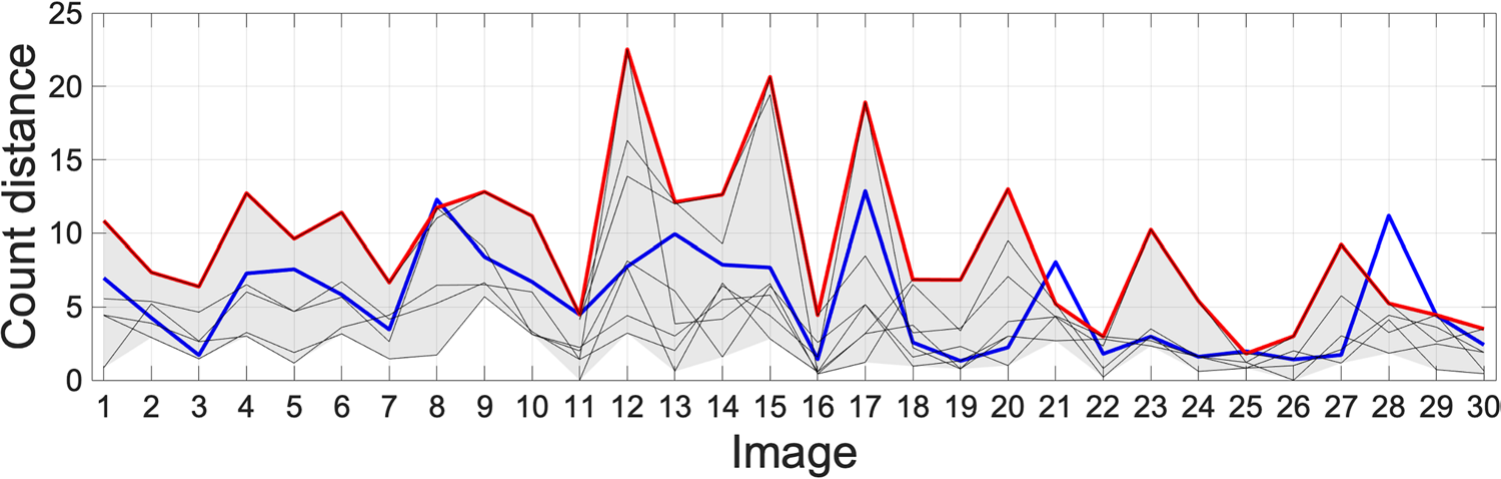
Distance from the centroid of the Ki67 and DAPI results from other counters. The maximum distance from the centroid of all researchers’ results is indicated in red. The distance from the centroid for the results from FINS is indicated in blue. The distance from the centroid for the results from each individual researcher is shown as grey lines. Images are from sets A–C

**Fig. 10:**
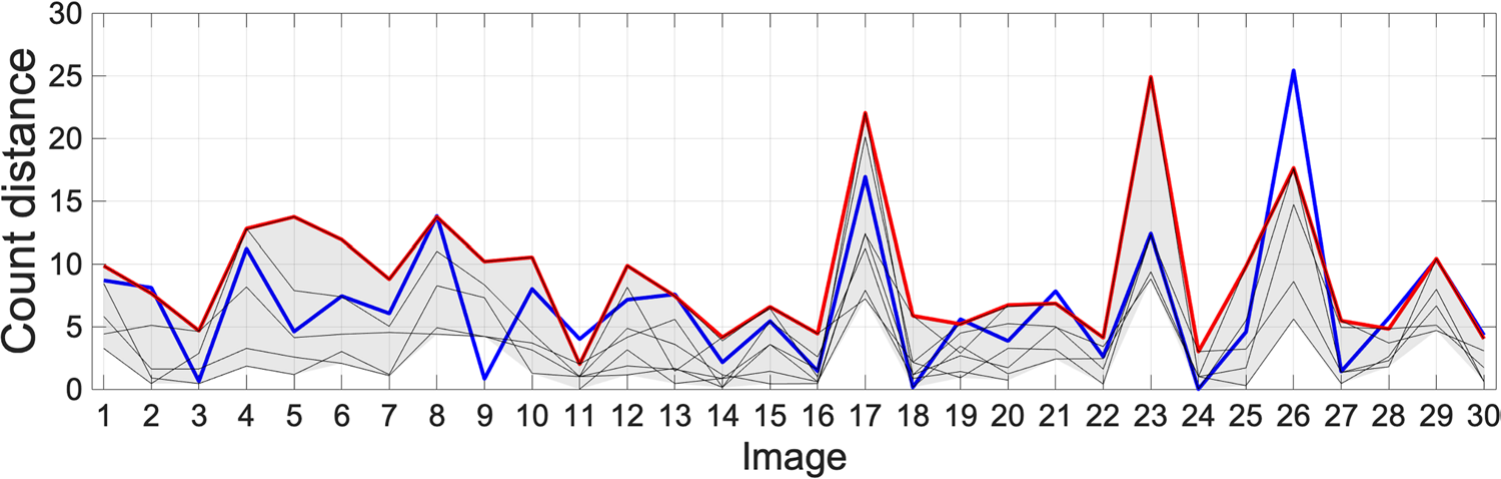
Distance from the centroid of the γH2AX and DAPI results from other counters. The maximum distance from the centroid of all researchers’ results is indicated in red. The distance from the centroid for the results from FINS is indicated in blue. The distances from the centroid for the results from each individual researcher are shown as grey lines. Images are from sets A–C

Counts from each channel are not useful in isolation, so we now investigate the performance of the algorithm in the context of these connections. We have introduced a quantitative measure of count similarity when Ki67 and γH2AX counts are related back to the DAPI count. In Figs. 7 and 8, we plot count data for DAPI versus Ki67 (set B) and γH2AX (set C), respectively. This shows a visual representation of how FINS counts compare against the researcher counts for these datasets. For each image, we computed the centroid of the researchers’ counts and calculated the distance each researcher’s count is from this point. We can then use the maximum of these distances to give a quantitative measure of concordance. The percentage concordance for FINS is then defined as the percentage of instances when the algorithm’s count distance from this centroid is below the maximum of the researchers’ distances. We can calculate a similar metric for each researcher, e.g., percentage concordance for researcher 1 would be based on distances from a centroid computed using counts from researchers 2–5. We should note that this does give FINS an inherent slight advantage with this metric as FINS compares against five counts (whereas the researchers compare against four counts) and the maximum distance (hence percentage concordance also) is likely to increase slightly when more counts are present. Nonetheless, it gives us a measure of the extent to which the algorithm counts are similar to the researchers’ counts. The results are presented in Table 1 and Figs. 9 and 10.

**Table 1:** Percentage concordance comparison between FINS and researcher counts for sets A, B, and C

Counter	Percentage concordance	
	γH2AX and DAPI	Ki67 and DAPI
Researcher 1	73.3	80.0
Researcher 2	70.0	76.7
Researcher 3	83.3	80.0
Researcher 4	20.0	16.7
Researcher 5	83.3	83.3
FINS	80.0	86.7

Percentage concordance is the percentage of instances that the counter’s results were outside the range of the other counter’s results. The results are based on association with either of the two proteins of interest and the DAPI count

The results from Table 1 support the idea that FINS count performance is similar to that of the researchers. A percentage concordance of 86.7 for DAPI versus Ki67 counts is higher than any of the researchers. For DAPI versus γH2AX counts, the percentage concordance is 80, with only researchers 3 and 5 higher at 83.3. Figures 9 and 10 show how these results are calculated for FINS. The blue line here is the distance of the FINS count from the centroid of the researchers’ counts. The maximum distance of the researchers’ data is indicated by the red line (the range is shaded grey). Here, we can visualise the consistency of the count data. It is worth stating that the maximum distance tends to be higher for set B due to having additional researcher data. When FINS is above this maximum distance, this is not to say that it is unacceptable in these cases. In the same way, a researcher count being furthest away from the centroid does not invalidate their data, FINS occasionally (as shown by the percentage concordance) being furthest away is not a negative. In fact, with the algorithm designed to function like a researcher count, we would expect it to be sometimes furthest away from the centroid.

We now include a visualisation of the results that highlight the relationship between DAPI and Ki67 counts; Fig. 7 shows the results for set B. It allows us to observe to what extent the results of the algorithm “cluster” with the manual counts using an important biological metric. Figure 7 shows that images 12, 13, 14, and 15 have data that is quite spread (although it should be noted that there are seven researchers’ counts for this data). This demonstrates the inherent variability in the data, highlighting the challenge of counting the cells consistently. We can see that the algorithm performance in these cases is reasonable based on the distances in Fig. 9, where FINS is below the maximum distance. For the remaining images, where the data are more clustered, the results are very strong. Over image sets A, B, and C, there are four cases where FINS is above the maximum distance. For images 8 and 21, they are over by less than 1. Images 21 and 28 are 2.85 and 5.95, respectively, over the maximum distance. For image 28, the researchers’ range for DAPI is 64–70, and for Ki67 is 2–10. The FINS count here is DAPI = 61 and Ki67 = 16. This particular image has 11 cells that are cropped by the border of the image. It is subjective as to whether or not to count them, and in this case, it appears most researchers have counted them. However, FINS has not counted them as the signal from them is not likely to have exceeded the threshold in the same way as the other cells in the image. This is arguably preferable because if a full nucleus cannot be observed and no nuclear protein staining is seen, we cannot be sure if the nuclear protein is stained but is located outside of the image border. Whilst the undercount of DAPI is acceptable, its appearance in tandem with the Ki67 overcount could be of concern. However, the Ki67 overcount appears to be explicable as the result of high levels of autofluorescence. High levels of background autofluorescence are discussed previously, and whilst FINS may not be able to deal with such irregularity in an image as well as a researcher could, it is more likely to be consistent when faced with a difficult image, and the risk of this is mitigated by the ability to review data with the user interface.

Similar results were also obtained for DAPI versus γH2AX counts for set C, shown in Fig. 8. Again, this is useful because it allows us to assess the similarity of the counts between manual acquisition and the proposed algorithm. Here, we can see that the FINS count clusters very tightly with the manual counts for the majority of images, confirmed by the distances in Fig. 10. These figures allow us to see clearly that the algorithm is very consistent for DAPI in these cases. Overall, the trend is very similar to the DAPI versus Ki67 comparison above. It is difficult to distinguish the algorithm’s performance from the manual data. There are anomalous cases, but this is true for the researchers’ data as well. For DAPI versus γH2AX (Fig. 8), there are six cases where FINS is above the maximum distance. Images 2, 8, 21, 28, and 30 are less than 1 over the maximum distance, indicating a trivial difference. However, image 26 is 7.79 over the limit. We can see from Fig. 10 that there is a near-consensus in the DAPI count (the FINS count is within the range of researchers’ counts), indicating that the error is primarily in the γH2AX count which is 54 for this image (the range of the researchers here is 11–46). However, FINS is closer to the counts of researchers 1, 3, 4, and 5 (mean count = 33) than researcher 2. This particular image has high levels of background autofluorescence meaning that it has inherently more subjectivity over which cell can be identified as being stained for γH2AX. This causes the wide variation in user counts but arguably proves the need for the algorithm: by its nature, it is more likely to behave consistently than researchers when faced with difficult images. Overall, these visualisations provide further support for the reliability of FINS.

### Time Comparisons

**Fig. 11:**
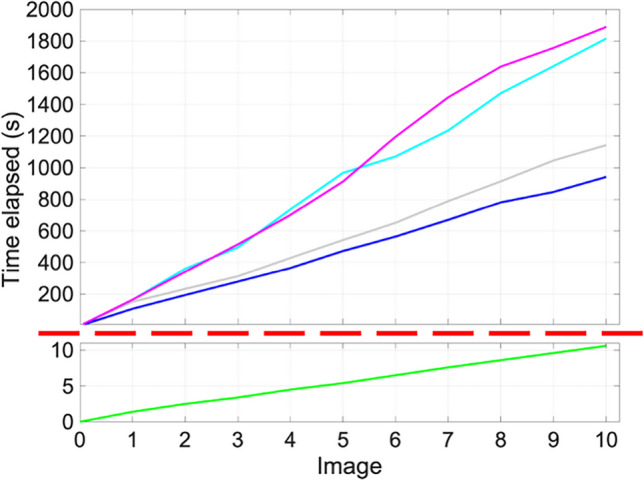
Time in seconds taken to analyse ten images from set A. The red dashed line indicates a break in the *y*-axis. Researchers 1–4 are indicated by grey, dark blue, light blue, and pink lines, respectively. The FINS algorithm is in green

We can see from Table 2 that the mean time per image for the algorithm is 1.06 s. Figure 11 shows this in comparison to researchers 1–4, with a split axis used because of the scales involved. It shows the variability of the time taken for each user and emphasises the difference between the algorithm and counting cells manually. These results are very positive in the sense that the algorithm takes approximately 1% of the time a manual process takes. Coupled with the accuracy performance discussed above, this is a potentially transformative development. Batches of images that would take hours to process now take minutes to get a result automatically.

**Table 2:** Summary of data for set A

Counter	Mean count per image			Mean time per image (s)
	Ki67	γH2AX	DAPI	
Researcher 1	12.1	21.0	60.0	104
Researcher 2	11.1	18.0	57.6	84.7
Researcher 3	9.40	17.9	59.3	164
Researcher 4	16.3	18.3	58.6	189
FINS	9.70	18.1	58.2	1.06

Mean cell count and time spent counting per image in seconds for ten images (*n* = 10)

## Discussion

The FINS algorithm is able to compete with manual counting methods in terms of accuracy and significantly reduces the time taken to analyse images whilst behaving consistently and more objectively. For images of this type, there are currently several methods for obtaining accurate count data. There is the option to count images manually (identifying cells to count by eye with or without the use of a click-counter/annotation tool), or there are image analysis platforms sold as part of expensive software suites, or free customisable image analysis pipelines. Although the nucleus itself can often be visualised using 4′,6-diamidino-2-phenylindole (DAPI) or similar stains and segmented easily using basic software techniques, it is more difficult to segment an image based on other stains, especially with the staining that is only useful when co-localised with the nuclear stain and may have multiple foci.

Images that are manually counted cannot be compared between researchers as there is often inconsistency; each researcher’s classification of a cell being stained for the protein of interest, or not, may suffer from researcher bias. Similarly, direct comparisons between image datasets from previous experiments/studies undertaken by different researchers may be problematic. Many of the reasons for this level of subjectivity relate to the individual user’s decision-making (manual determination of what constitutes a cell/signal and whether or not to count it), experience with immunocytochemistry/manual cell counting, eyesight, tiredness, care, and focus. There are also differences in how researchers perform manual counts—whether they export images to an alternative format, if they use a tool or not for counting and/or annotation. There are also technical factors that can affect the data, e.g., the screen being used or the level of ambient light at the time of the count. Before analysis can begin, there is already variation introduced between images caused by the researcher’s choices regarding adjustment of any image capture settings, not to mention the biological and technical variation that comes with any tissue culture and immunocytochemical staining techniques. All manual counting techniques are time-consuming and can be tiring for the researcher, but manually counting cells does mean that researchers can check the images for any irregularities that might not be identified with an automated image analysis tool. There is also inherent biological and technical variability with this type of staining experiment. For example, irregularities could be present such as an instance of two cells overlaid following mitosis and higher levels of background autofluorescence in an image or cell debris. In this particular biological application within our field of research, nuclear size can often vary considerably within an image of the same cell type. Senescent cells can exhibit polyploidy with very large nuclei as a result, and any cells undergoing mitosis can give condensed small nuclei [14]. In this context, attempting to be more stringent to avoid counting cell debris would cause the omission of valuable cell count data, so a compromise is sought. Although an automated image analysis tool would not necessarily be able to compensate for all irregularities, it would still remove a lot of subjectivity from the image analysis method compared to manual counts. Ideally, any alternative software would have a user interface to enable checking for irregularities such as those mentioned above.

In spite of the problems with manual counting, many researchers still choose to do so. Many academic research groups do not perform enough of this type of analysis to warrant the expensive purchase of software suites such as the HALO® Image Analysis Platform (Indica Labs Inc., Albuquerque, NM, USA) and many do not have the bioinformatic skills necessary to create, perfect, and optimise a custom pipeline using software such as QuPath or Cell Profiler™ [15, 16]. There are also indications that a fully custom-built algorithm performs better than approaches built using custom pipeline software [17]. Although some algorithms built for another purpose (e.g., for analysis of tissue sections that are immunohistochemically stained) may be easily adaptable for assessment of immunocytochemically stained images for a bioinformatician, they may be difficult to find as they are often niche, and it is not often feasible for an end-point researcher to spend time finding, adapting, and validating it for their own purpose. Therefore, many academic studies still tend to use manually acquired data despite the approach’s severe limitations.

For an algorithm to be viable as an alternative to manual counting, its data must be comparable to an average researcher’s counts. The FINS algorithm has very good percentage concordance scores in Table 1 and appears to be similar to the researchers. Although the FINS algorithm may appear to often be at the edge of the range of the counts in Figs. 4, 5, and 6, there are mitigating factors as discussed previously, and when the counts are put into context to look at the proportion of cells that are stained for a protein of interest (Figs. 8, 9, and 10 and Table 1), the algorithm is similar to the individual researchers with how often it is at the edge of the range of the researchers’ counts. Being at the edge of the range for raw counts, but not for proportion, data is perhaps down to nuances in how a researcher may deal with an image differently, e.g., some researchers may not count cells that are at the border of images meaning that their data is consistent with other researchers’ data for the proportion of cells stained for the protein of interest despite their differing raw counts. In fact, FINS’ percentage concordance with the researcher counts is above the median of the individual researchers’ scores for both proteins of interest and is the most consistent for Ki67.

The main advantage of replacing manual counts with an algorithm is the time saved in acquiring the data. In Table 2, we present the mean time taken to manually count an image, which ranges from 84.7 to 189 s. This is a significant drain on time and resources given that image datasets in this field are often large, and the count data is crucial to drawing meaningful conclusions. Although we present a small dataset here, experimental datasets are often significantly larger. For example, the average number of images per dataset produced by one researcher was 195 images. This would correspond to 4 h and 35 min if the researcher was (unrealistically) able to continuously count non-stop at the fastest researcher’s normal speed. A dataset of that size would more realistically take a few working days to complete. It is worth noting that the counting time for this dataset was of reasonable duration to allow an individual to retain focus. It is likely that with a larger dataset, the time per image would increase and/or time for a break would have to be included. Despite only having data for set A, it is certainly enough to give an impression of how long manual counts take for this type of data.

Another key advantage of using an algorithm is to reduce the workload of the users, giving more time for analysis over acquisition. This does imply that the computation time of the algorithm is irrelevant; the user can run the algorithm and return to inspect the results at a later time. However, this is not the ideal approach in this case. Our aim was to create a framework where the results could be supervised in near real-time, such that the counts could be efficiently and reliably acquired. There was therefore a requirement to keep the computation time per image as low as possible.

Here, we built a simple tool that can be run using Matlab software (MathWorks Inc., Natick, MA, USA) that is omnipresent in academic research and familiar to many researchers. Our tool is designed specifically for the analysis of images of immunocytochemically stained nuclear proteins. The tool also has the capability to review data (the user interface is shown in Fig. 2) and can be adjusted/adapted for other parameters/software according to the needs of the individual researcher, but its primary aim is simplicity of use. The script is simple, easily accessible, and free, so we hope that the barriers stopping people from using other methods do not apply to this algorithm.

## Conclusion

The algorithm demonstrates that its accuracy is comparable to that of manual counting. It is more consistent and less subjective than manual counting due to the nature of the algorithm. When examining small biological effects, they can be masked by variability caused by inconsistencies in the method of counting stained cells. The algorithm can reproduce its counts and is able to apply the same parameters to each image, unlike researchers who are inherently more variable in their counting approach. The algorithm also allows for better comparison between datasets; all datasets must be counted by the same researcher in order to make the same comparison, which is often unfeasible. The time saving brought about by the use of the algorithm represents a key benefit in itself. The time savings could also allow researchers to perform more experiments/replicates or image more cells, which could provide greater statistical power for the identification of any small biological differences in an experiment. This algorithm could also be applied to fluorescent staining for other general nuclear stains (e.g., Hoescht), nuclear proteins of interest (e.g., RNA splicing factors), and for assaying nuclear components indirectly (e.g., the TUNEL assay, terminal deoxynucleotidyl transferase dUTP nick end labelling of DNA breaks). Whilst other custom pipeline programs, commercial programs, or algorithms may exist that could be adapted to produce similar results, we believe this algorithm to be useful as it is quick, easy, simple, and free to run, with the ability to review images if needed. These qualities make it attractive for a non-programmer specialist/general laboratory scientist over manual counting and other alternatives.

## Supplementary Information

Below is the link to the electronic supplementary material.

Supplementary file1 (PDF 361 KB)
