# Supplementary material for: Image Analysis Using the Fluorescence Imaging of Nuclear Staining (FINS) Algorithm
Source: J Imaging Inform Med. 2024 Jun 17;37(6):3077–89. doi: 10.1007/s10278-024-01097-8 (PMC11641597; doi:10.1007/s10278-024-01097-8)
Supplement: Supplementary file 1 — Supplementary file1 (PDF 361 KB) [file 10278_2024_1097_MOESM1_ESM.pdf]

```
function varargout = v2_0_gui(varargin)

gui_Singleton = 1;
gui_State = struct('gui_Name',       mfilename, ...
                   'gui_Singleton',  gui_Singleton, ...
                   'gui_OpeningFcn', @v2_0_gui_OpeningFcn, ...
                   'gui_OutputFcn',  @v2_0_gui_OutputFcn, ...
                   'gui_LayoutFcn',  [] , ...
                   'gui_Callback',   []);
if nargin && ischar(varargin{1})
    gui_State.gui_Callback = str2func(varargin{1});
end

if nargout
    [varargout{1:nargout}] = gui_mainfcn(gui_State, varargin{:});
else
    gui_mainfcn(gui_State, varargin{:});
end
% End initialization code - DO NOT EDIT

% --- Executes just before v2_0_gui is made visible.
function v2_0_gui_OpeningFcn(hObject, eventdata, handles, varargin)

global z0 zed DZ DUB DUR DUG CP numObj_r numObj_b numObj_g liffile

tic
CF = pwd; AP = fullfile(CF,'pro_images');
addpath(AP)
I0 = dir(fullfile(AP,'*.lif'));
liffile = I0.name;
filename=fullfile(AP,I0.name);
IMG = JS_loadLif2(filename);

[n,ig] = size(IMG);

handles.popupmenu_images.String = cell(n,1);
for k = 1:n
  name = IMG{k,1};
  handles.popupmenu_images.String{k} = name;
end

d1 = 512; d2 = 512;
zed = zeros(d1,d2);

D00 = cell(n,8);
for i = 1:n
z10 = IMG{i,2}; z20 = IMG{i,3}; z30 = IMG{i,4};
rz1 = rot90(z10,3); z1 = fliplr(rz1);
rz2 = rot90(z20,3); z2 = fliplr(rz2);
rz3 = rot90(z30,3); z3 = fliplr(rz3);
Z = cat(3,z3,z2,z1);
z0 = imresize(Z,[d1,d2]);
D00(i,1) = {z0}; D00(i,8) = IMG(i,1);
D0 = Cells_v5(D00,z0,zed,i);
D00 = D0;
```

```
time = toc;
fprintf('Count complete for Image %d/%d, %1.1f seconds \n',i,n,time);
end
CP = D0;

[a,b] = size(D0);
rd = zeros(a,1)'; rh = zeros(a,1)'; rk = zeros(a,1)';
for j = 1:a
    rd(j) = D0{j,3}; rh(j) = D0{j,7}; rk(j) = D0{j,5};
end

clear D00 D0

set(handles.UB_checkbox,'value',1)
set(handles.UR_checkbox,'value',1)
set(handles.UG_checkbox,'value',1)

im_no = get(handles.popupmenu_images,'Value');
DZ = CP{im_no,1};

% Display variables
z0 = DZ; Im = z0;
DUB = CP{im_no,2}; numObj_b = CP{im_no,3};
DUR = CP{im_no,4}; numObj_r = CP{im_no,5};
DUG = CP{im_no,6}; numObj_g = CP{im_no,7};

CP{im_no,7};

% warning suppression
warning('off','MATLAB:contour:ConstantData');
warning('off','images:multithresh:degenerateInput');

cla
imshow(Im);caxis([0 255]);axis off;
caption = sprintf('DAPI# = %d, Ki67# = %d, H2AX# = %d', numObj_b,numObj_r,numObj_g);
    title(caption, 'FontSize', 14);
hold on;
contour(DUR,[0.5 0.5],'color',[1 0 0],'LineWidth',1);
contour(DUB,[0.5 0.5],'color',[0 0 1],'LineWidth',1);
contour(DUG,[0.5 0.5],'color',[0 1 0],'LineWidth',1);
hold off;

% Choose default command line output for v2_0_gui
handles.output = hObject;

% Update handles structure
guidata(hObject, handles);

% --- Outputs from this function are returned to the command line.
function varargout = v2_0_gui_OutputFcn(hObject, eventdata, handles)
% varargout  cell array for returning output args (see VARARGOUT);
% hObject    handle to figure
% eventdata  reserved - to be defined in a future version of MATLAB
% handles    structure with handles and user data (see GUIDATA)
```

```
% Get default command line output from handles structure
varargout{1} = handles.output;

function bw_checkbox_Callback(hObject, eventdata, handles)

global DZ DUB DUR DUG bwmax numObj_r numObj_b numObj_g

val_bw = get(handles.bw_checkbox, 'Value');

if val_bw == 1
   I0 = rgb2gray(DZ); bwmax = max(I0(:));
   Im = I0;
else
   Im = DZ; bwmax = 255;
end
if bwmax < 50
    bwmax = 50;
end

cla
imshow(Im);caxis([0 bwmax]);axis off;
caption = sprintf('DAPI# = %d, Ki67# = %d, H2AX# = %d', numObj_b,numObj_r,numObj_g);
    title(caption, 'FontSize', 14);
hold on;
contour(DUR,[0.5 0.5],'color',[1 0 0],'LineWidth',1);
contour(DUB,[0.5 0.5],'color',[0 0 1],'LineWidth',1);
contour(DUG,[0.5 0.5],'color',[0 1 0],'LineWidth',1);
hold off;

function ch1_checkbox_Callback(hObject, eventdata, handles)

global zed DZ z0 DUB DUR DUG bwmax numObj_r numObj_b numObj_g

val_ch1 = get(handles.ch1_checkbox, 'Value'); val_bw = get(handles.bw_checkbox,↙
'Value');

if val_ch1 == 1
   DZ(:,:,1) = z0(:,:,1);
else
   DZ(:,:,1) = zed;
end

if val_bw == 1
   I0 = rgb2gray(DZ); bwmax = max(I0(:));
   Im = I0;
else
   Im = DZ; bwmax = 255;
end
if bwmax < 50
    bwmax = 50;
end

cla
imshow(Im);caxis([0 bwmax]);axis off;
caption = sprintf('DAPI# = %d, Ki67# = %d, H2AX# = %d', numObj_b,numObj_r,numObj_g);
```

```
    title(caption, 'FontSize', 14);
hold on;
contour(DUR,[0.5 0.5],'color',[1 0 0],'LineWidth',1);
contour(DUB,[0.5 0.5],'color',[0 0 1],'LineWidth',1);
contour(DUG,[0.5 0.5],'color',[0 1 0],'LineWidth',1);
hold off;

function ch2_checkbox_Callback(hObject, eventdata, handles)

global zed DZ z0 DUB DUR DUG bwmax numObj_r numObj_b numObj_g

val_ch2 = get(handles.ch2_checkbox, 'Value'); val_bw = get(handles.bw_checkbox,↙
'Value');

if val_ch2 == 1
   DZ(:,:,2) = z0(:,:,2);
else
   DZ(:,:,2) = zed;
end

if val_bw == 1
   I0 = rgb2gray(DZ); bwmax = max(I0(:));
   Im = I0;
else
   Im = DZ; bwmax = 255;
end
if bwmax < 50
    bwmax = 50;
end

cla
imshow(Im);caxis([0 bwmax]);axis off;
caption = sprintf('DAPI# = %d, Ki67# = %d, H2AX# = %d', numObj_b,numObj_r,numObj_g);
    title(caption, 'FontSize', 14);
hold on;
contour(DUR,[0.5 0.5],'color',[1 0 0],'LineWidth',1);
contour(DUB,[0.5 0.5],'color',[0 0 1],'LineWidth',1);
contour(DUG,[0.5 0.5],'color',[0 1 0],'LineWidth',1);
hold off;

function ch3_checkbox_Callback(hObject, eventdata, handles)

global z0 zed DZ DUB DUR DUG bwmax numObj_r numObj_b numObj_g

val_ch3 = get(handles.ch3_checkbox, 'Value'); val_bw = get(handles.bw_checkbox,↙
'Value');

if val_ch3 == 1
   DZ(:,:,3) = z0(:,:,3);
else
   DZ(:,:,3) = zed;
end

if val_bw == 1
   I0 = rgb2gray(DZ); bwmax = max(I0(:));
```

```
   Im = I0;
else
   Im = DZ; bwmax = 255;
end
if bwmax < 50
    bwmax = 50;
end

cla
imshow(Im);caxis([0 bwmax]);axis off;
caption = sprintf('DAPI# = %d, Ki67# = %d, H2AX# = %d', numObj_b,numObj_r,numObj_g);
    title(caption, 'FontSize', 14);
hold on;
contour(DUR,[0.5 0.5],'color',[1 0 0],'LineWidth',1);
contour(DUB,[0.5 0.5],'color',[0 0 1],'LineWidth',1);
contour(DUG,[0.5 0.5],'color',[0 1 0],'LineWidth',1);
hold off;

function UB_checkbox_Callback(hObject, eventdata, handles)

global zed DUB DUR DUG DZ bwmax numObj_r numObj_b numObj_g CP

im_no = handles.popupmenu_images.Value;
val_ub = get(handles.UB_checkbox, 'Value');
val_bw = get(handles.bw_checkbox, 'Value');

if val_ub == 1
   DUB = CP{im_no,2};
else
   DUB = zed;
end

if val_bw == 1
   I0 = rgb2gray(DZ); bwmax = max(I0(:));
   Im = I0;
else
   Im = DZ; bwmax = 255;
end
if bwmax < 50
    bwmax = 50;
end

cla
imshow(Im);caxis([0 bwmax]);axis off;
caption = sprintf('DAPI# = %d, Ki67# = %d, H2AX# = %d', numObj_b,numObj_r,numObj_g);
    title(caption, 'FontSize', 14);
hold on;
contour(DUR,[0.5 0.5],'color',[1 0 0],'LineWidth',1);
contour(DUB,[0.5 0.5],'color',[0 0 1],'LineWidth',1);
contour(DUG,[0.5 0.5],'color',[0 1 0],'LineWidth',1);
hold off;

function UR_checkbox_Callback(hObject, eventdata, handles)

global zed DUR DUB DUG DZ bwmax numObj_r numObj_b numObj_g CP
```

```
im_no = handles.popupmenu_images.Value;
val_ur = get(handles.UR_checkbox, 'Value');
val_bw = get(handles.bw_checkbox, 'Value');

if val_ur == 1
   DUR = CP{im_no,4};
else
   DUR = zed;
end

if val_bw == 1
   I0 = rgb2gray(DZ); bwmax = max(I0(:));
   Im = I0;
else
   Im = DZ; bwmax = 255;
end
if bwmax < 50
    bwmax = 50;
end

cla
imshow(Im);caxis([0 bwmax]);axis off;
caption = sprintf('DAPI# = %d, Ki67# = %d, H2AX# = %d', numObj_b,numObj_r,numObj_g);
    title(caption, 'FontSize', 14);
hold on;
contour(DUR,[0.5 0.5],'color',[1 0 0],'LineWidth',1);
contour(DUB,[0.5 0.5],'color',[0 0 1],'LineWidth',1);
contour(DUG,[0.5 0.5],'color',[0 1 0],'LineWidth',1);
hold off;

% --- Executes on button press in UG_checkbox.
function UG_checkbox_Callback(hObject, eventdata, handles)

global zed DUR DUB DUG DZ bwmax numObj_r numObj_b numObj_g CP

im_no = handles.popupmenu_images.Value;
val_ug = get(handles.UG_checkbox, 'Value');
val_bw = get(handles.bw_checkbox, 'Value');

if val_ug == 1
   DUG = CP{im_no,6};
else
   DUG = zed;
end

if val_bw == 1
   I0 = rgb2gray(DZ); bwmax = max(I0(:));
   Im = I0;
else
   Im = DZ; bwmax = 255;
end
if bwmax < 50
    bwmax = 50;
end
```

```
cla
imshow(Im);caxis([0 bwmax]);axis off;
caption = sprintf('DAPI# = %d, Ki67# = %d, H2AX# = %d', numObj_b,numObj_r,numObj_g);
    title(caption, 'FontSize', 14);
hold on;
contour(DUR,[0.5 0.5],'color',[1 0 0],'LineWidth',1);
contour(DUB,[0.5 0.5],'color',[0 0 1],'LineWidth',1);
contour(DUG,[0.5 0.5],'color',[0 1 0],'LineWidth',1);
hold off;

% --- Executes on selection change in popupmenu_images.
function popupmenu_images_Callback(hObject, eventdata, handles)

global CP z0 DZ DUB DUR DUG numObj_b numObj_r numObj_g

set(handles.bw_checkbox,'value',0)
set(handles.ch1_checkbox,'value',1)
set(handles.ch2_checkbox,'value',1)
set(handles.ch3_checkbox,'value',1)
set(handles.UB_checkbox,'value',1)
set(handles.UR_checkbox,'value',1)
set(handles.UG_checkbox,'value',1)

im_no = handles.popupmenu_images.Value;

z0 = CP{im_no,1};
DUB = CP{im_no,2}; numObj_b = CP{im_no,3};
DUR = CP{im_no,4}; numObj_r = CP{im_no,5};
DUG = CP{im_no,6}; numObj_g = CP{im_no,7};

DZ = CP{im_no,1};
Im = DZ;

cla
imshow(Im);caxis([0 255]);axis off;
caption = sprintf('DAPI# = %d, Ki67# = %d, H2AX# = %d', numObj_b,numObj_r,numObj_g);
    title(caption, 'FontSize', 14);
hold on;
contour(DUR,[0.5 0.5],'color',[1 0 0],'LineWidth',1);
contour(DUB,[0.5 0.5],'color',[0 0 1],'LineWidth',1);
contour(DUG,[0.5 0.5],'color',[0 1 0],'LineWidth',1);
hold off;

% --- Executes during object creation, after setting all properties.
function popupmenu_images_CreateFcn(hObject, eventdata, handles)
% hObject    handle to popupmenu_images (see GCBO)
% eventdata  reserved - to be defined in a future version of MATLAB
% handles    empty - handles not created until after all CreateFcns called

if ispc && isequal(get(hObject,'BackgroundColor'), get↙
(0,'defaultUicontrolBackgroundColor'))
    set(hObject,'BackgroundColor','white');
end
```

```
% --- Executes on button press in pushbutton_save.
function pushbutton_save_Callback(hObject, eventdata, handles)
% hObject    handle to pushbutton_save (see GCBO)
% eventdata  reserved - to be defined in a future version of MATLAB
% handles    structure with handles and user data (see GUIDATA)

global CP liffile

[a1,a2] = size(CP); m = cell(a1+3,3);
t = now; d = datetime(t,'ConvertFrom','datenum');
d0 = date; ds = datestr(d);

m{2,1} = '21 Nov 2022'; m{3,1} = 'Image name';
m{2,2} = 'V2.0'; m{3,2} = 'DAPI count';
m{2,3} = ds; m{3,3} = 'H2AX count';
m{2,4} = liffile; m{3,4} = 'Ki67 count';

for k = 1:a1
m{k+3,1} = CP{k,8}; % image name
m{k+3,2} = CP{k,3}; m{k+3,3} = CP{k,7}; m{k+3,4} = CP{k,5};
end

T = cell2table(m(2:end,:),'VariableNames',{'ICC_Seg' 'Version' 'Date_Time' 'File'});

str0 = sprintf('ICC_V2.0_%d_count_',a1); str1 = [str0,d0]; str2 = '.csv';
str = [str1,str2];

writetable(T,str)

function D0 = Cells_v5(D00,z0,zed,i)

lambda_b = 20; tf = 0.1;
%%%%%%%%%%%%% START PUSH BLUE
B = im2double(z0(:,:,3));
thresh = multithresh(B);
b = B - thresh;
fb = -b;
U = Lsegv1_Run(lambda_b,fb);
U0 = zed; U0(U>0.5) = 1;
[lIm, numObj_b] = bwlabel(U0);
D00(i,2) = {U0}; D00(i,3) = {numObj_b};
UB = U0;
%%% END PUSH BLUE
%%% PRELIMS
cc = bwconncomp(UB);
ObN = regionprops(cc);
[n,ig] = size(ObN);
UR = zed; UG = zed; count_r = 0; count_g = 0; % pre-allocate
b0 = bwdist(UB); BD0 = zed; BD0(b0<2) = 1;
vg0 = 0.15; vr0 = 0.12;
%%% END
R = im2double(z0(:,:,1)); G = im2double(z0(:,:,2));
rb = R.*UB; nr = nonzeros(rb); vr = multithresh(nr);
if vr < tf, vr = vr0; end
gb = G.*UB; ng = nonzeros(gb);
```

```
vg2 = multithresh(ng,2); vg = vg2(1);
if vg < tf, vg = vg0; end
for j = 1:n
w = ObN(j).BoundingBox;
a1 = round(w(1)); a2 = round(w(3)); b1 = round(w(2)); b2 = round(w(4));
qb = UB(b1:(b1+b2-1),a1:(a1+a2-1));
%%% red count
qr = R(b1:(b1+b2-1),a1:(a1+a2-1));
xr = qb.*qr;
if max(xr(:)) > vr
   count_r = count_r + 1;
   UR(b1:(b1+b2-1),a1:(a1+a2-1)) = BD0(b1:(b1+b2-1),a1:(a1+a2-1));
end
%%% green count
qg = G(b1:(b1+b2-1),a1:(a1+a2-1));
xg = qb.*qg;
if max(xg(:)) > vg
   count_g = count_g + 1;
   UG(b1:(b1+b2-1),a1:(a1+a2-1)) = BD0(b1:(b1+b2-1),a1:(a1+a2-1));
end
end
numObj_r = count_r; numObj_g = count_g;
D00(i,4) = {UR}; D00(i,5) = {numObj_r};
D00(i,6) = {UG}; D00(i,7) = {numObj_g};
%%% END
D0 = D00;

function U = Lsegv1_Run(lambda,f)

[d1,d2] = size(f);
U = zeros(d1,d2);
u = SB_ATV(f,1/lambda);
U(u<0)=1;

function u = SB_ATV(g,mu)
% Split Bregman Anisotropic Total Variation Denoising
% u = arg min_u 1/2||u-g||_2^2 + mu*ATV(u)

g = g(:);
n = length(g);
[B,Bt,BtB] = DiffOper(sqrt(n));
b = zeros(2*n,1);
d = b;
u = g;
err = 1;k = 1;
tol = 1e-3;
lambda = 1;
while err > tol
    up = u;
    [u,~] = cgs(speye(n)+BtB, g-lambda*Bt*(b-d),1e-5,100);
    Bub = B*u+b;
    d = max(abs(Bub)-mu/lambda,0).*sign(Bub);
    b = Bub-d;
    err = norm(up-u)/norm(u);
    k = k+1;
```

```
end

function [B,Bt,BtB] = DiffOper(N)
D = spdiags([-ones(N,1) ones(N,1)], [0 1], N,N+1);
D(:,1) = [];
D(1,1) = 0;
B = [ kron(speye(N),D) ; kron(D,speye(N)) ];
Bt = B';
BtB = Bt*B;
```

```
function varargout = v2_0_gui_export(varargin)

gui_Singleton = 1;
gui_State = struct('gui_Name',       mfilename, ...
                   'gui_Singleton',  gui_Singleton, ...
                   'gui_OpeningFcn', @v2_0_gui_export_OpeningFcn, ...
                   'gui_OutputFcn',  @v2_0_gui_export_OutputFcn, ...
                   'gui_LayoutFcn',  @v2_0_gui_export_LayoutFcn, ...
                   'gui_Callback',   []);
if nargin && ischar(varargin{1})
    gui_State.gui_Callback = str2func(varargin{1});
end

if nargout
    [varargout{1:nargout}] = gui_mainfcn(gui_State, varargin{:});
else
    gui_mainfcn(gui_State, varargin{:});
end
% End initialization code - DO NOT EDIT

% --- Executes just before v2_0_gui_export is made visible.
function v2_0_gui_export_OpeningFcn(hObject, eventdata, handles, varargin)

global z0 zed DZ DUB DUR DUG CP numObj_r numObj_b numObj_g liffile

tic
CF = pwd; AP = fullfile(CF,'pro_images');
addpath(AP)
I0 = dir(fullfile(AP,'*.lif'));
liffile = I0.name;
filename=fullfile(AP,I0.name);
IMG = JS_loadLif2(filename);

[n,ig] = size(IMG);

handles.popupmenu_images.String = cell(n,1);
for k = 1:n
  name = IMG{k,1};
  handles.popupmenu_images.String{k} = name;
end

d1 = 512; d2 = 512;
zed = zeros(d1,d2);

D00 = cell(n,8);
for i = 1:n
z10 = IMG{i,2}; z20 = IMG{i,3}; z30 = IMG{i,4};
rz1 = rot90(z10,3); z1 = fliplr(rz1);
rz2 = rot90(z20,3); z2 = fliplr(rz2);
rz3 = rot90(z30,3); z3 = fliplr(rz3);
Z = cat(3,z3,z2,z1);
z0 = imresize(Z,[d1,d2]);
D00(i,1) = {z0}; D00(i,8) = IMG(i,1);
D0 = Cells_v5(D00,z0,zed,i);
D00 = D0;
```

```
time = toc;
fprintf('Count complete for Image %d/%d, %1.1f seconds \n',i,n,time);
end
CP = D0;

[a,b] = size(D0);
rd = zeros(a,1)'; rh = zeros(a,1)'; rk = zeros(a,1)';
for j = 1:a
    rd(j) = D0{j,3}; rh(j) = D0{j,7}; rk(j) = D0{j,5};
end

clear D00 D0

set(handles.UB_checkbox,'value',1)
set(handles.UR_checkbox,'value',1)
set(handles.UG_checkbox,'value',1)

im_no = get(handles.popupmenu_images,'Value');
DZ = CP{im_no,1};

% Display variables
z0 = DZ; Im = z0;
DUB = CP{im_no,2}; numObj_b = CP{im_no,3};
DUR = CP{im_no,4}; numObj_r = CP{im_no,5};
DUG = CP{im_no,6}; numObj_g = CP{im_no,7};

CP{im_no,7};

% warning suppression
warning('off','MATLAB:contour:ConstantData');
warning('off','images:multithresh:degenerateInput');

cla
imshow(Im);caxis([0 255]);axis off;
caption = sprintf('DAPI# = %d, Ki67# = %d, H2AX# = %d', numObj_b,numObj_r,numObj_g);
    title(caption, 'FontSize', 14);
hold on;
contour(DUR,[0.5 0.5],'color',[1 0 0],'LineWidth',1);
contour(DUB,[0.5 0.5],'color',[0 0 1],'LineWidth',1);
contour(DUG,[0.5 0.5],'color',[0 1 0],'LineWidth',1);
hold off;

% Choose default command line output for v2_0_gui_export
handles.output = hObject;

% Update handles structure
guidata(hObject, handles);

% --- Outputs from this function are returned to the command line.
function varargout = v2_0_gui_export_OutputFcn(hObject, eventdata, handles)
% varargout  cell array for returning output args (see VARARGOUT);
% hObject    handle to figure
% eventdata  reserved - to be defined in a future version of MATLAB
% handles    structure with handles and user data (see GUIDATA)
```

```
% Get default command line output from handles structure
varargout{1} = handles.output;

function bw_checkbox_Callback(hObject, eventdata, handles)

global DZ DUB DUR DUG bwmax numObj_r numObj_b numObj_g

val_bw = get(handles.bw_checkbox, 'Value');

if val_bw == 1
   I0 = rgb2gray(DZ); bwmax = max(I0(:));
   Im = I0;
else
   Im = DZ; bwmax = 255;
end
if bwmax < 50
    bwmax = 50;
end

cla
imshow(Im);caxis([0 bwmax]);axis off;
caption = sprintf('DAPI# = %d, Ki67# = %d, H2AX# = %d', numObj_b,numObj_r,numObj_g);
    title(caption, 'FontSize', 14);
hold on;
contour(DUR,[0.5 0.5],'color',[1 0 0],'LineWidth',1);
contour(DUB,[0.5 0.5],'color',[0 0 1],'LineWidth',1);
contour(DUG,[0.5 0.5],'color',[0 1 0],'LineWidth',1);
hold off;

function ch1_checkbox_Callback(hObject, eventdata, handles)

global zed DZ z0 DUB DUR DUG bwmax numObj_r numObj_b numObj_g

val_ch1 = get(handles.ch1_checkbox, 'Value'); val_bw = get(handles.bw_checkbox,↙
'Value');

if val_ch1 == 1
   DZ(:,:,1) = z0(:,:,1);
else
   DZ(:,:,1) = zed;
end

if val_bw == 1
   I0 = rgb2gray(DZ); bwmax = max(I0(:));
   Im = I0;
else
   Im = DZ; bwmax = 255;
end
if bwmax < 50
    bwmax = 50;
end

cla
imshow(Im);caxis([0 bwmax]);axis off;
caption = sprintf('DAPI# = %d, Ki67# = %d, H2AX# = %d', numObj_b,numObj_r,numObj_g);
```

```
    title(caption, 'FontSize', 14);
hold on;
contour(DUR,[0.5 0.5],'color',[1 0 0],'LineWidth',1);
contour(DUB,[0.5 0.5],'color',[0 0 1],'LineWidth',1);
contour(DUG,[0.5 0.5],'color',[0 1 0],'LineWidth',1);
hold off;

function ch2_checkbox_Callback(hObject, eventdata, handles)

global zed DZ z0 DUB DUR DUG bwmax numObj_r numObj_b numObj_g

val_ch2 = get(handles.ch2_checkbox, 'Value'); val_bw = get(handles.bw_checkbox,↙
'Value');

if val_ch2 == 1
   DZ(:,:,2) = z0(:,:,2);
else
   DZ(:,:,2) = zed;
end

if val_bw == 1
   I0 = rgb2gray(DZ); bwmax = max(I0(:));
   Im = I0;
else
   Im = DZ; bwmax = 255;
end
if bwmax < 50
    bwmax = 50;
end

cla
imshow(Im);caxis([0 bwmax]);axis off;
caption = sprintf('DAPI# = %d, Ki67# = %d, H2AX# = %d', numObj_b,numObj_r,numObj_g);
    title(caption, 'FontSize', 14);
hold on;
contour(DUR,[0.5 0.5],'color',[1 0 0],'LineWidth',1);
contour(DUB,[0.5 0.5],'color',[0 0 1],'LineWidth',1);
contour(DUG,[0.5 0.5],'color',[0 1 0],'LineWidth',1);
hold off;

function ch3_checkbox_Callback(hObject, eventdata, handles)

global z0 zed DZ DUB DUR DUG bwmax numObj_r numObj_b numObj_g

val_ch3 = get(handles.ch3_checkbox, 'Value'); val_bw = get(handles.bw_checkbox,↙
'Value');

if val_ch3 == 1
   DZ(:,:,3) = z0(:,:,3);
else
   DZ(:,:,3) = zed;
end

if val_bw == 1
   I0 = rgb2gray(DZ); bwmax = max(I0(:));
```

```
   Im = I0;
else
   Im = DZ; bwmax = 255;
end
if bwmax < 50
    bwmax = 50;
end

cla
imshow(Im);caxis([0 bwmax]);axis off;
caption = sprintf('DAPI# = %d, Ki67# = %d, H2AX# = %d', numObj_b,numObj_r,numObj_g);
    title(caption, 'FontSize', 14);
hold on;
contour(DUR,[0.5 0.5],'color',[1 0 0],'LineWidth',1);
contour(DUB,[0.5 0.5],'color',[0 0 1],'LineWidth',1);
contour(DUG,[0.5 0.5],'color',[0 1 0],'LineWidth',1);
hold off;

function UB_checkbox_Callback(hObject, eventdata, handles)

global zed DUB DUR DUG DZ bwmax numObj_r numObj_b numObj_g CP

im_no = handles.popupmenu_images.Value;
val_ub = get(handles.UB_checkbox, 'Value');
val_bw = get(handles.bw_checkbox, 'Value');

if val_ub == 1
   DUB = CP{im_no,2};
else
   DUB = zed;
end

if val_bw == 1
   I0 = rgb2gray(DZ); bwmax = max(I0(:));
   Im = I0;
else
   Im = DZ; bwmax = 255;
end
if bwmax < 50
    bwmax = 50;
end

cla
imshow(Im);caxis([0 bwmax]);axis off;
caption = sprintf('DAPI# = %d, Ki67# = %d, H2AX# = %d', numObj_b,numObj_r,numObj_g);
    title(caption, 'FontSize', 14);
hold on;
contour(DUR,[0.5 0.5],'color',[1 0 0],'LineWidth',1);
contour(DUB,[0.5 0.5],'color',[0 0 1],'LineWidth',1);
contour(DUG,[0.5 0.5],'color',[0 1 0],'LineWidth',1);
hold off;

function UR_checkbox_Callback(hObject, eventdata, handles)

global zed DUR DUB DUG DZ bwmax numObj_r numObj_b numObj_g CP
```

```
im_no = handles.popupmenu_images.Value;
val_ur = get(handles.UR_checkbox, 'Value');
val_bw = get(handles.bw_checkbox, 'Value');

if val_ur == 1
   DUR = CP{im_no,4};
else
   DUR = zed;
end

if val_bw == 1
   I0 = rgb2gray(DZ); bwmax = max(I0(:));
   Im = I0;
else
   Im = DZ; bwmax = 255;
end
if bwmax < 50
    bwmax = 50;
end

cla
imshow(Im);caxis([0 bwmax]);axis off;
caption = sprintf('DAPI# = %d, Ki67# = %d, H2AX# = %d', numObj_b,numObj_r,numObj_g);
    title(caption, 'FontSize', 14);
hold on;
contour(DUR,[0.5 0.5],'color',[1 0 0],'LineWidth',1);
contour(DUB,[0.5 0.5],'color',[0 0 1],'LineWidth',1);
contour(DUG,[0.5 0.5],'color',[0 1 0],'LineWidth',1);
hold off;

% --- Executes on button press in UG_checkbox.
function UG_checkbox_Callback(hObject, eventdata, handles)

global zed DUR DUB DUG DZ bwmax numObj_r numObj_b numObj_g CP

im_no = handles.popupmenu_images.Value;
val_ug = get(handles.UG_checkbox, 'Value');
val_bw = get(handles.bw_checkbox, 'Value');

if val_ug == 1
   DUG = CP{im_no,6};
else
   DUG = zed;
end

if val_bw == 1
   I0 = rgb2gray(DZ); bwmax = max(I0(:));
   Im = I0;
else
   Im = DZ; bwmax = 255;
end
if bwmax < 50
    bwmax = 50;
end
```

```
cla
imshow(Im);caxis([0 bwmax]);axis off;
caption = sprintf('DAPI# = %d, Ki67# = %d, H2AX# = %d', numObj_b,numObj_r,numObj_g);
    title(caption, 'FontSize', 14);
hold on;
contour(DUR,[0.5 0.5],'color',[1 0 0],'LineWidth',1);
contour(DUB,[0.5 0.5],'color',[0 0 1],'LineWidth',1);
contour(DUG,[0.5 0.5],'color',[0 1 0],'LineWidth',1);
hold off;

% --- Executes on selection change in popupmenu_images.
function popupmenu_images_Callback(hObject, eventdata, handles)

global CP z0 DZ DUB DUR DUG numObj_b numObj_r numObj_g

set(handles.bw_checkbox,'value',0)
set(handles.ch1_checkbox,'value',1)
set(handles.ch2_checkbox,'value',1)
set(handles.ch3_checkbox,'value',1)
set(handles.UB_checkbox,'value',1)
set(handles.UR_checkbox,'value',1)
set(handles.UG_checkbox,'value',1)

im_no = handles.popupmenu_images.Value;

z0 = CP{im_no,1};
DUB = CP{im_no,2}; numObj_b = CP{im_no,3};
DUR = CP{im_no,4}; numObj_r = CP{im_no,5};
DUG = CP{im_no,6}; numObj_g = CP{im_no,7};

DZ = CP{im_no,1};
Im = DZ;

cla
imshow(Im);caxis([0 255]);axis off;
caption = sprintf('DAPI# = %d, Ki67# = %d, H2AX# = %d', numObj_b,numObj_r,numObj_g);
    title(caption, 'FontSize', 14);
hold on;
contour(DUR,[0.5 0.5],'color',[1 0 0],'LineWidth',1);
contour(DUB,[0.5 0.5],'color',[0 0 1],'LineWidth',1);
contour(DUG,[0.5 0.5],'color',[0 1 0],'LineWidth',1);
hold off;

% --- Executes during object creation, after setting all properties.
function popupmenu_images_CreateFcn(hObject, eventdata, handles)
% hObject    handle to popupmenu_images (see GCBO)
% eventdata  reserved - to be defined in a future version of MATLAB
% handles    empty - handles not created until after all CreateFcns called

if ispc && isequal(get(hObject,'BackgroundColor'), get↙
(0,'defaultUicontrolBackgroundColor'))
    set(hObject,'BackgroundColor','white');
end
```

```
% --- Executes on button press in pushbutton_save.
function pushbutton_save_Callback(hObject, eventdata, handles)
% hObject    handle to pushbutton_save (see GCBO)
% eventdata  reserved - to be defined in a future version of MATLAB
% handles    structure with handles and user data (see GUIDATA)

global CP liffile

[a1,a2] = size(CP); m = cell(a1+3,3);
t = now; d = datetime(t,'ConvertFrom','datenum');
d0 = date; ds = datestr(d);

m{2,1} = '21 Nov 2022'; m{3,1} = 'Image name';
m{2,2} = 'V2.0'; m{3,2} = 'DAPI count';
m{2,3} = ds; m{3,3} = 'H2AX count';
m{2,4} = liffile; m{3,4} = 'Ki67 count';

for k = 1:a1
m{k+3,1} = CP{k,8}; % image name
m{k+3,2} = CP{k,3}; m{k+3,3} = CP{k,7}; m{k+3,4} = CP{k,5};
end

T = cell2table(m(2:end,:),'VariableNames',{'ICC_Seg' 'Version' 'Date_Time' 'File'});

str0 = sprintf('ICC_V2.0_%d_count_',a1); str1 = [str0,d0]; str2 = '.csv';
str = [str1,str2];

writetable(T,str)

function D0 = Cells_v5(D00,z0,zed,i)

lambda_b = 20; tf = 0.1;
%%%%%%%%%%%%% START PUSH BLUE
B = im2double(z0(:,:,3));
thresh = multithresh(B);
b = B - thresh;
fb = -b;
U = Lsegv1_Run(lambda_b,fb);
U0 = zed; U0(U>0.5) = 1;
[lIm, numObj_b] = bwlabel(U0);
D00(i,2) = {U0}; D00(i,3) = {numObj_b};
UB = U0;
%%% END PUSH BLUE
%%% PRELIMS
cc = bwconncomp(UB);
ObN = regionprops(cc);
[n,ig] = size(ObN);
UR = zed; UG = zed; count_r = 0; count_g = 0; % pre-allocate
b0 = bwdist(UB); BD0 = zed; BD0(b0<2) = 1;
vg0 = 0.15; vr0 = 0.12;
%%% END
R = im2double(z0(:,:,1)); G = im2double(z0(:,:,2));
rb = R.*UB; nr = nonzeros(rb); vr = multithresh(nr);
if vr < tf, vr = vr0; end
gb = G.*UB; ng = nonzeros(gb);
```

```
vg2 = multithresh(ng,2); vg = vg2(1);
if vg < tf, vg = vg0; end
for j = 1:n
w = ObN(j).BoundingBox;
a1 = round(w(1)); a2 = round(w(3)); b1 = round(w(2)); b2 = round(w(4));
qb = UB(b1:(b1+b2-1),a1:(a1+a2-1));
%%% red count
qr = R(b1:(b1+b2-1),a1:(a1+a2-1));
xr = qb.*qr;
if max(xr(:)) > vr
   count_r = count_r + 1;
   UR(b1:(b1+b2-1),a1:(a1+a2-1)) = BD0(b1:(b1+b2-1),a1:(a1+a2-1));
end
%%% green count
qg = G(b1:(b1+b2-1),a1:(a1+a2-1));
xg = qb.*qg;
if max(xg(:)) > vg
   count_g = count_g + 1;
   UG(b1:(b1+b2-1),a1:(a1+a2-1)) = BD0(b1:(b1+b2-1),a1:(a1+a2-1));
end
end
numObj_r = count_r; numObj_g = count_g;
D00(i,4) = {UR}; D00(i,5) = {numObj_r};
D00(i,6) = {UG}; D00(i,7) = {numObj_g};
%%% END
D0 = D00;

function U = Lsegv1_Run(lambda,f)

[d1,d2] = size(f);
U = zeros(d1,d2);
u = SB_ATV(f,1/lambda);
U(u<0)=1;

function u = SB_ATV(g,mu)
% Split Bregman Anisotropic Total Variation Denoising
% u = arg min_u 1/2||u-g||_2^2 + mu*ATV(u)

g = g(:);
n = length(g);
[B,Bt,BtB] = DiffOper(sqrt(n));
b = zeros(2*n,1);
d = b;
u = g;
err = 1;k = 1;
tol = 1e-3;
lambda = 1;
while err > tol
    up = u;
    [u,~] = cgs(speye(n)+BtB, g-lambda*Bt*(b-d),1e-5,100);
    Bub = B*u+b;
    d = max(abs(Bub)-mu/lambda,0).*sign(Bub);
    b = Bub-d;
    err = norm(up-u)/norm(u);
    k = k+1;
```

```
end

function [B,Bt,BtB] = DiffOper(N)
D = spdiags([-ones(N,1) ones(N,1)], [0 1], N,N+1);
D(:,1) = [];
D(1,1) = 0;
B = [ kron(speye(N),D) ; kron(D,speye(N)) ];
Bt = B';
BtB = Bt*B;


% --- Creates and returns a handle to the GUI figure.
function h1 = v2_0_gui_export_LayoutFcn(policy)
% policy - create a new figure or use a singleton. 'new' or 'reuse'.

persistent hsingleton;
if strcmpi(policy, 'reuse') & ishandle(hsingleton)
    h1 = hsingleton;
    return;
end
load v2_0_gui_export.mat


appdata = [];
appdata.GUIDEOptions = struct(...
    'active_h', [], ...
    'taginfo', struct(...
    'figure', 2, ...
    'text', 2, ...
    'axes', 2, ...
    'uipanel', 3, ...
    'popupmenu', 3, ...
    'slider', 2, ...
    'pushbutton', 5, ...
    'checkbox', 8, ...
    'listbox', 2, ...
    'uitoolbar', 2, ...
    'uitoggletool', 5), ...
    'override', 0, ...
    'release', [], ...
    'resize', 'none', ...
    'accessibility', 'callback', ...
    'mfile', 1, ...
    'callbacks', 1, ...
    'singleton', 1, ...
    'syscolorfig', 1, ...
    'blocking', 0, ...
    'lastSavedFile', 'D:\FINS Algorithm\ICCv2\v2_0_gui_export.m', ...
    'lastFilename', 'D:\FINS Algorithm\ICCv2\v2_0_gui.fig');
appdata.lastValidTag = 'figure1';
appdata.GUIDELayoutEditor = [];
appdata.initTags = struct(...
    'handle', [], ...
    'tag', 'figure1');
```

```
h1 = figure(...
'PaperUnits','inches',...
'Units','characters',...
'Position',[135.8 17.5384615384615 260 57.6153846153846],...
'Visible',get(0,'defaultfigureVisible'),...
'Color',get(0,'defaultfigureColor'),...
'CurrentAxesMode','manual',...
'IntegerHandle','off',...
'Colormap',get(0,'defaultfigureColormap'),...
'MenuBar','none',...
'Name','v2_0_gui',...
'NumberTitle','off',...
'HandleVisibility','callback',...
'Tag','figure1',...
'DockControls',get(0,'defaultfigureDockControls'),...
'Resize','off',...
'PaperPosition',get(0,'defaultfigurePaperPosition'),...
'PaperSize',[8.5 11],...
'PaperSizeMode',get(0,'defaultfigurePaperSizeMode'),...
'PaperType','usletter',...
'PaperTypeMode',get(0,'defaultfigurePaperTypeMode'),...
'PaperUnitsMode',get(0,'defaultfigurePaperUnitsMode'),...
'InvertHardcopy','off',...
'ScreenPixelsPerInchMode','manual',...
'CreateFcn', {@local_CreateFcn, blanks(0), appdata} );

appdata = [];
appdata.lastValidTag = 'axes1';

h2 = axes(...
'Parent',h1,...
'FontUnits',get(0,'defaultaxesFontUnits'),...
'Units','characters',...
'CameraPosition',[0.5 0.5 9.16025403784439],...
'CameraPositionMode',get(0,'defaultaxesCameraPositionMode'),...
'CameraTarget',[0.5 0.5 0.5],...
'CameraTargetMode',get(0,'defaultaxesCameraTargetMode'),...
'CameraViewAngle',6.60861036031192,...
'CameraViewAngleMode',get(0,'defaultaxesCameraViewAngleMode'),...
'PlotBoxAspectRatio',[1 0.981099656357388 0.981099656357388],...
'PlotBoxAspectRatioMode',get(0,'defaultaxesPlotBoxAspectRatioMode'),...
'Colormap',[0.2422 0.1504 0.6603;0.250390476190476 0.164995238095238↙
0.707614285714286;0.257771428571429 0.181780952380952 0.751138095238095;↙
0.264728571428571 0.197757142857143 0.795214285714286;0.270647619047619↙
0.21467619047619 0.836371428571429;0.275114285714286 0.234238095238095↙
0.870985714285714;0.2783 0.255871428571429 0.899071428571429;0.280333333333333↙
0.278233333333333 0.9221;0.281338095238095 0.300595238095238 0.941376190476191;↙
0.281014285714286 0.322757142857143 0.957885714285714;0.279466666666667↙
0.344671428571429 0.971676190476191;0.275971428571429 0.366680952380952↙
0.982904761904762;0.269914285714286 0.3892 0.9906;0.260242857142857 0.412328571428571↙
0.995157142857143;0.244033333333333 0.435833333333333 0.998833333333333;↙
0.220642857142857 0.460257142857143 0.997285714285714;0.196333333333333↙
0.484719047619048 0.989152380952381;0.183404761904762 0.507371428571429↙
0.979795238095238;0.178642857142857 0.528857142857143 0.968157142857143;↙
0.176438095238095 0.549904761904762 0.952019047619048;0.168742857142857↙
```

```
0.570261904761905 0.935871428571428;0.154 0.5902 0.9218;0.146028571428571↙
0.609119047619048 0.907857142857143;0.13802380952381 0.627628571428572↙
0.897290476190476;0.124814285714286 0.645928571428571 0.888342857142857;↙
0.111252380952381 0.6635 0.876314285714286;0.0952095238095238 0.679828571428571↙
0.859780952380952;0.0688714285714285 0.694771428571429 0.839357142857143;↙
0.0296666666666667 0.708166666666667 0.816333333333333;0.00357142857142857↙
0.720266666666667 0.7917;0.00665714285714287 0.731214285714286 0.766014285714286;↙
0.0433285714285715 0.741095238095238 0.739409523809524;0.096395238095238 0.75↙
0.712038095238095;0.140771428571429 0.7584 0.684157142857143;0.1717 0.766961904761905↙
0.655442857142857;0.193766666666667 0.775766666666667 0.6251;0.216085714285714 0.7843↙
0.5923;0.246957142857143 0.791795238095238 0.556742857142857;0.290614285714286↙
0.797290476190476 0.518828571428572;0.340642857142857 0.8008 0.478857142857143;0.3909↙
0.802871428571428 0.435447619047619;0.445628571428572 0.802419047619048↙
0.390919047619048;0.5044 0.7993 0.348;0.561561904761905 0.794233333333333↙
0.304480952380953;0.617395238095238 0.787619047619048 0.261238095238095;↙
0.671985714285714 0.779271428571429 0.2227;0.7242 0.769842857142857 0.191028571428571;↙
0.773833333333333 0.759804761904762 0.164609523809524;0.820314285714286↙
0.749814285714286 0.153528571428571;0.863433333333333 0.7406 0.159633333333333;↙
0.903542857142857 0.733028571428571 0.177414285714286;0.939257142857143↙
0.728785714285714 0.209957142857143;0.972757142857143 0.729771428571429↙
0.239442857142857;0.995647619047619 0.743371428571429 0.237147619047619;↙
0.996985714285714 0.765857142857143 0.219942857142857;0.995204761904762↙
0.789252380952381 0.202761904761905;0.9892 0.813566666666667 0.188533333333333;↙
0.978628571428571 0.838628571428572 0.176557142857143;0.967647619047619 0.8639↙
0.164290476190476;0.961009523809524 0.889019047619048 0.153676190476191;↙
0.959671428571429 0.913457142857143 0.142257142857143;0.962795238095238↙
0.937338095238095 0.126509523809524;0.969114285714286 0.960628571428571↙
0.106361904761905;0.9769 0.9839 0.0805],...
'ColormapMode',get(0,'defaultaxesColormapMode'),...
'Alphamap',[0 0.0159 0.0317 0.0476 0.0635 0.0794 0.0952 0.1111 0.127 0.1429 0.1587↙
0.1746 0.1905 0.2063 0.2222 0.2381 0.254 0.2698 0.2857 0.3016 0.3175 0.3333 0.3492↙
0.3651 0.381 0.3968 0.4127 0.4286 0.4444 0.4603 0.4762 0.4921 0.5079 0.5238 0.5397↙
0.5556 0.5714 0.5873 0.6032 0.619 0.6349 0.6508 0.6667 0.6825 0.6984 0.7143 0.7302↙
0.746 0.7619 0.7778 0.7937 0.8095 0.8254 0.8413 0.8571 0.873 0.8889 0.9048 0.9206↙
0.9365 0.9524 0.9683 0.9841 1],...
'AlphamapMode',get(0,'defaultaxesAlphamapMode'),...
'XTick',[0 0.1 0.2 0.3 0.4 0.5 0.6 0.7 0.8 0.9 1],...
'XTickMode',get(0,'defaultaxesXTickMode'),...
'XTickLabel',{  '0'; '0.1'; '0.2'; '0.3'; '0.4'; '0.5'; '0.6'; '0.7'; '0.8'; '0.9';↙
'1' },...
'XTickLabelMode',get(0,'defaultaxesXTickLabelMode'),...
'YTick',[0 0.1 0.2 0.3 0.4 0.5 0.6 0.7 0.8 0.9 1],...
'YTickMode',get(0,'defaultaxesYTickMode'),...
'YTickLabel',{  '0'; '0.1'; '0.2'; '0.3'; '0.4'; '0.5'; '0.6'; '0.7'; '0.8'; '0.9';↙
'1' },...
'YTickLabelMode',get(0,'defaultaxesYTickLabelMode'),...
'CameraMode',get(0,'defaultaxesCameraMode'),...
'DataSpaceMode',get(0,'defaultaxesDataSpaceMode'),...
'ColorSpaceMode',get(0,'defaultaxesColorSpaceMode'),...
'DecorationContainerMode',get(0,'defaultaxesDecorationContainerMode'),...
'ChildContainerMode',get(0,'defaultaxesChildContainerMode'),...
'XRulerMode',get(0,'defaultaxesXRulerMode'),...
'YRulerMode',get(0,'defaultaxesYRulerMode'),...
'ZRulerMode',get(0,'defaultaxesZRulerMode'),...
'AmbientLightSourceMode',get(0,'defaultaxesAmbientLightSourceMode'),...
```

```
'Position',[7 11.174358974359 116.428571428571 43.8666666666667],...
'ActivePositionProperty','position',...
'ActivePositionPropertyMode',get(0,'defaultaxesActivePositionPropertyMode'),...
'LooseInset',[33.8 6.33769230769231 24.7 4.32115384615384],...
'LooseInsetMode',get(0,'defaultaxesLooseInsetMode'),...
'ColorOrder',get(0,'defaultaxesColorOrder'),...
'SortMethod','childorder',...
'SortMethodMode',get(0,'defaultaxesSortMethodMode'),...
'Tag','axes1',...
'CreateFcn', {@local_CreateFcn, blanks(0), appdata} );

h3 = get(h2,'title');

set(h3,...
'Parent',h2,...
'Units','data',...
'FontUnits','points',...
'DecorationContainer',[],...
'DecorationContainerMode','auto',...
'Color',[0 0 0],...
'ColorMode','auto',...
'Position',[0.500000535827322 1.00481611208406 0.500000000000007],...
'PositionMode','auto',...
'Interpreter','tex',...
'InterpreterMode','auto',...
'Rotation',0,...
'RotationMode','auto',...
'FontName','Helvetica',...
'FontNameMode','auto',...
'FontUnitsMode','auto',...
'FontSize',11,...
'FontSizeMode','auto',...
'FontAngle','normal',...
'FontAngleMode','auto',...
'FontWeight','bold',...
'FontWeightMode','auto',...
'HorizontalAlignment','center',...
'HorizontalAlignmentMode','auto',...
'VerticalAlignment','bottom',...
'VerticalAlignmentMode','auto',...
'EdgeColor','none',...
'EdgeColorMode','auto',...
'LineStyle','-',...
'LineStyleMode','auto',...
'LineWidth',0.5,...
'LineWidthMode','auto',...
'BackgroundColor','none',...
'BackgroundColorMode','auto',...
'Margin',3,...
'MarginMode','auto',...
'Clipping','off',...
'ClippingMode','auto',...
'Layer','middle',...
'LayerMode','auto',...
'FontSmoothing','on',...
```

```
'FontSmoothingMode','auto',...
'UnitsMode','auto',...
'IncludeRenderer','on',...
'IsContainer','off',...
'IsContainerMode','auto',...
'DimensionNames',{  'X' 'Y' 'Z' },...
'DimensionNamesMode','auto',...
'XLimInclude','on',...
'XLimIncludeMode','auto',...
'YLimInclude','on',...
'YLimIncludeMode','auto',...
'ZLimInclude','on',...
'ZLimIncludeMode','auto',...
'CLimInclude','on',...
'CLimIncludeMode','auto',...
'ALimInclude','on',...
'ALimIncludeMode','auto',...
'Description','Axes Title',...
'DescriptionMode','auto',...
'Visible','on',...
'VisibleMode','auto',...
'Serializable','on',...
'SerializableMode','auto',...
'HandleVisibility','off',...
'HandleVisibilityMode','auto',...
'TransformForPrintFcnImplicitInvoke','on',...
'TransformForPrintFcnImplicitInvokeMode','auto',...
'HG1EraseMode','auto',...
'BusyAction','queue',...
'Interruptible','on',...
'HitTest','on',...
'HitTestMode','auto',...
'PickableParts','visible',...
'PickablePartsMode','auto');

h4 = get(h2,'xlabel');

set(h4,...
'Parent',h2,...
'Units','data',...
'FontUnits','points',...
'DecorationContainer',[],...
'DecorationContainerMode','auto',...
'Color',[0.15 0.15 0.15],...
'ColorMode','auto',...
'Position',[0.500000476837158 -0.0414477515937429 7.105427357601e-15],...
'PositionMode','auto',...
'Interpreter','tex',...
'InterpreterMode','auto',...
'Rotation',0,...
'RotationMode','auto',...
'FontName','Helvetica',...
'FontNameMode','auto',...
'FontUnitsMode','auto',...
'FontSize',11,...
```

```
'FontSizeMode','auto',...
'FontAngle','normal',...
'FontAngleMode','auto',...
'FontWeight','normal',...
'FontWeightMode','auto',...
'HorizontalAlignment','center',...
'HorizontalAlignmentMode','auto',...
'VerticalAlignment','top',...
'VerticalAlignmentMode','auto',...
'EdgeColor','none',...
'EdgeColorMode','auto',...
'LineStyle','-',...
'LineStyleMode','auto',...
'LineWidth',0.5,...
'LineWidthMode','auto',...
'BackgroundColor','none',...
'BackgroundColorMode','auto',...
'Margin',3,...
'MarginMode','auto',...
'Clipping','off',...
'ClippingMode','auto',...
'Layer','back',...
'LayerMode','auto',...
'FontSmoothing','on',...
'FontSmoothingMode','auto',...
'UnitsMode','auto',...
'IncludeRenderer','on',...
'IsContainer','off',...
'IsContainerMode','auto',...
'DimensionNames',{  'X'  'Y'  'Z' },...
'DimensionNamesMode','auto',...
'XLimInclude','on',...
'XLimIncludeMode','auto',...
'YLimInclude','on',...
'YLimIncludeMode','auto',...
'ZLimInclude','on',...
'ZLimIncludeMode','auto',...
'CLimInclude','on',...
'CLimIncludeMode','auto',...
'ALimInclude','on',...
'ALimIncludeMode','auto',...
'Description','AxisRulerBase Label',...
'DescriptionMode','auto',...
'Visible','on',...
'VisibleMode','auto',...
'Serializable','on',...
'SerializableMode','auto',...
'HandleVisibility','off',...
'HandleVisibilityMode','auto',...
'TransformForPrintFcnImplicitInvoke','on',...
'TransformForPrintFcnImplicitInvokeMode','auto',...
'HG1EraseMode','auto',...
'BusyAction','queue',...
'Interruptible','on',...
'HitTest','on',...
```

```
'HitTestMode','auto',...
'PickableParts','visible',...
'PickablePartsMode','auto');

h5 = get(h2,'ylabel');

set(h5,...
'Parent',h2,...
'Units','data',...
'FontUnits','points',...
'DecorationContainer',[],...
'DecorationContainerMode','auto',...
'Color',[0.15 0.15 0.15],...
'ColorMode','auto',...
'Position',[-0.0486827023489506 0.500000476837158 7.105427357601e-15],...
'PositionMode','auto',...
'Interpreter','tex',...
'InterpreterMode','auto',...
'Rotation',90,...
'RotationMode','auto',...
'FontName','Helvetica',...
'FontNameMode','auto',...
'FontUnitsMode','auto',...
'FontSize',11,...
'FontSizeMode','auto',...
'FontAngle','normal',...
'FontAngleMode','auto',...
'FontWeight','normal',...
'FontWeightMode','auto',...
'HorizontalAlignment','center',...
'HorizontalAlignmentMode','auto',...
'VerticalAlignment','bottom',...
'VerticalAlignmentMode','auto',...
'EdgeColor','none',...
'EdgeColorMode','auto',...
'LineStyle','-',...
'LineStyleMode','auto',...
'LineWidth',0.5,...
'LineWidthMode','auto',...
'BackgroundColor','none',...
'BackgroundColorMode','auto',...
'Margin',3,...
'MarginMode','auto',...
'Clipping','off',...
'ClippingMode','auto',...
'Layer','back',...
'LayerMode','auto',...
'FontSmoothing','on',...
'FontSmoothingMode','auto',...
'UnitsMode','auto',...
'IncludeRenderer','on',...
'IsContainer','off',...
'IsContainerMode','auto',...
'DimensionNames',{  'X' 'Y' 'Z' },...
'DimensionNamesMode','auto',...
```

```
'XLimInclude','on',...
'XLimIncludeMode','auto',...
'YLimInclude','on',...
'YLimIncludeMode','auto',...
'ZLimInclude','on',...
'ZLimIncludeMode','auto',...
'CLimInclude','on',...
'CLimIncludeMode','auto',...
'ALimInclude','on',...
'ALimIncludeMode','auto',...
'Description','AxisRulerBase Label',...
'DescriptionMode','auto',...
'Visible','on',...
'VisibleMode','auto',...
'Serializable','on',...
'SerializableMode','auto',...
'HandleVisibility','off',...
'HandleVisibilityMode','auto',...
'TransformForPrintFcnImplicitInvoke','on',...
'TransformForPrintFcnImplicitInvokeMode','auto',...
'HG1EraseMode','auto',...
'BusyAction','queue',...
'Interruptible','on',...
'HitTest','on',...
'HitTestMode','auto',...
'PickableParts','visible',...
'PickablePartsMode','auto');

h6 = get(h2,'zlabel');

set(h6,...
'Parent',h2,...
'Units','data',...
'FontUnits','points',...
'DecorationContainer',[],...
'DecorationContainerMode','auto',...
'Color',[0.15 0.15 0.15],...
'ColorMode','auto',...
'Position',[0 0 0],...
'PositionMode','auto',...
'Interpreter','tex',...
'InterpreterMode','auto',...
'Rotation',0,...
'RotationMode','auto',...
'FontName','Helvetica',...
'FontNameMode','auto',...
'FontUnitsMode','auto',...
'FontSize',10,...
'FontSizeMode','auto',...
'FontAngle','normal',...
'FontAngleMode','auto',...
'FontWeight','normal',...
'FontWeightMode','auto',...
'HorizontalAlignment','left',...
'HorizontalAlignmentMode','auto',...
```

```
'VerticalAlignment','middle',...
'VerticalAlignmentMode','auto',...
'EdgeColor','none',...
'EdgeColorMode','auto',...
'LineStyle','-',...
'LineStyleMode','auto',...
'LineWidth',0.5,...
'LineWidthMode','auto',...
'BackgroundColor','none',...
'BackgroundColorMode','auto',...
'Margin',3,...
'MarginMode','auto',...
'Clipping','off',...
'ClippingMode','auto',...
'Layer','middle',...
'LayerMode','auto',...
'FontSmoothing','on',...
'FontSmoothingMode','auto',...
'UnitsMode','auto',...
'IncludeRenderer','on',...
'IsContainer','off',...
'IsContainerMode','auto',...
'DimensionNames',{  'X' 'Y' 'Z' },...
'DimensionNamesMode','auto',...
'XLimInclude','on',...
'XLimIncludeMode','auto',...
'YLimInclude','on',...
'YLimIncludeMode','auto',...
'ZLimInclude','on',...
'ZLimIncludeMode','auto',...
'CLimInclude','on',...
'CLimIncludeMode','auto',...
'ALimInclude','on',...
'ALimIncludeMode','auto',...
'Description','AxisRulerBase Label',...
'DescriptionMode','auto',...
'Visible','off',...
'VisibleMode','auto',...
'Serializable','on',...
'SerializableMode','auto',...
'HandleVisibility','off',...
'HandleVisibilityMode','auto',...
'TransformForPrintFcnImplicitInvoke','on',...
'TransformForPrintFcnImplicitInvokeMode','auto',...
'HG1EraseMode','auto',...
'BusyAction','queue',...
'Interruptible','on',...
'HitTest','on',...
'HitTestMode','auto',...
'PickableParts','visible',...
'PickablePartsMode','auto');

appdata = [];
appdata.lastValidTag = 'uipanel1';
```

```
h7 = uipanel(...
'Parent',h1,...
'FontUnits',get(0,'defaultuipanelFontUnits'),...
'Units','characters',...
'Title','Data',...
'Tag','uipanel1',...
'Position',[17.4285714285714 5.37435897435904 96.7142857142857 5.8],...
'Layout',[],...
'CreateFcn', {@local_CreateFcn, blanks(0), appdata} );

appdata = [];
appdata.lastValidTag = 'pushbutton_save';

h8 = uicontrol(...
'Parent',h7,...
'Units','characters',...
'FontUnits',get(0,'defaultuicontrolFontUnits'),...
'HorizontalAlignment',get(0,'defaultuicontrolHorizontalAlignment'),...
'ListboxTop',get(0,'defaultuicontrolListboxTop'),...
'Max',get(0,'defaultuicontrolMax'),...
'Min',get(0,'defaultuicontrolMin'),...
'SliderStep',get(0,'defaultuicontrolSliderStep'),...
'String','Save Count',...
'Style',get(0,'defaultuicontrolStyle'),...
'Value',get(0,'defaultuicontrolValue'),...
'Position',[46.5714285714286 2.4 21.5714285714286 2.6],...
'BackgroundColor',get(0,'defaultuicontrolBackgroundColor'),...
'Callback',@(hObject,eventdata)v2_0_gui_export('pushbutton_save_Callback',hObject,↙
eventdata,guidata(hObject)),...
'Children',[],...
'ForegroundColor',get(0,'defaultuicontrolForegroundColor'),...
'Enable',get(0,'defaultuicontrolEnable'),...
'TooltipString',blanks(0),...
'Visible',get(0,'defaultuicontrolVisible'),...
'HandleVisibility',get(0,'defaultuicontrolHandleVisibility'),...
'ButtonDownFcn',blanks(0),...
'CreateFcn', {@local_CreateFcn, blanks(0), appdata} ,...
'DeleteFcn',blanks(0),...
'Tag','pushbutton_save',...
'UserData',[],...
'KeyPressFcn',blanks(0),...
'KeyReleaseFcn',blanks(0),...
'FontSize',14,...
'FontName',get(0,'defaultuicontrolFontName'),...
'FontAngle',get(0,'defaultuicontrolFontAngle'),...
'FontWeight',get(0,'defaultuicontrolFontWeight'));

appdata = [];
appdata.lastValidTag = 'UB_checkbox';

h9 = uicontrol(...
'Parent',h7,...
'Units','characters',...
'FontUnits',get(0,'defaultuicontrolFontUnits'),...
'String','Show DAPI Annotations',...
```

```
'Style','checkbox',...
'Position',[2.42857142857143 0.6 21.5714285714286 1.8],...
'Callback',@(hObject,eventdata)v2_0_gui_export('UB_checkbox_Callback',hObject,↙
eventdata,guidata(hObject)),...
'Children',[],...
'CreateFcn', {@local_CreateFcn, blanks(0), appdata} ,...
'Tag','UB_checkbox',...
'FontSize',10,...
'FontSizeMode',get(0,'defaultuicontrolFontSizeMode'));

appdata = [];
appdata.lastValidTag = 'UR_checkbox';

h10 = uicontrol(...
'Parent',h7,...
'Units','characters',...
'FontUnits',get(0,'defaultuicontrolFontUnits'),...
'String','Show Ki67 Annotations',...
'Style','checkbox',...
'Position',[25.1428571428571 0.6 21.1428571428571 1.8],...
'Callback',@(hObject,eventdata)v2_0_gui_export('UR_checkbox_Callback',hObject,↙
eventdata,guidata(hObject)),...
'Children',[],...
'ButtonDownFcn',blanks(0),...
'CreateFcn', {@local_CreateFcn, blanks(0), appdata} ,...
'DeleteFcn',blanks(0),...
'Tag','UR_checkbox',...
'KeyPressFcn',blanks(0),...
'FontSize',10,...
'FontSizeMode',get(0,'defaultuicontrolFontSizeMode'));

appdata = [];
appdata.lastValidTag = 'popupmenu_images';

h11 = uicontrol(...
'Parent',h7,...
'Units','characters',...
'FontUnits',get(0,'defaultuicontrolFontUnits'),...
'String',{  'Werner''s P10 A1 Overlay 1'; 'Werner''s P10 A1 Overlay 2'; 'Werner''s P10↙
A1 Overlay 3'; 'Werner''s P10 A1 Overlay 4'; 'Werner''s P10 A1 Overlay 5'; 'Werner''s↙
P10 B1 Overlay 1'; 'Werner''s P10 B1 Overlay 2'; 'Werner''s P10 B1 Overlay 3';↙
'Werner''s P10 B1 Overlay 4'; 'Werner''s P10 B1 Overlay 5'; 'Werner''s P10 C1 Overlay↙
1'; 'Werner''s P10 C1 Overlay 2'; 'Werner''s P10 C1 Overlay 3'; 'Werner''s P10 C1↙
Overlay 4'; 'Werner''s P10 C1 Overlay 5'; 'Werner''s P13 A1 Overlay 1'; 'Werner''s P13↙
A1 Overlay 2'; 'Werner''s P13 A1 Overlay 3'; 'Werner''s P13 A1 Overlay 4'; 'Werner''s↙
P13 A1 Overlay 5'; 'Werner''s P13 A2 Overlay 1'; 'Werner''s P13 A2 Overlay 3';↙
'Werner''s P13 A2 Overlay 4'; 'Werner''s P13 A2 Overlay 5'; 'Werner''s P13 A3 Overlay↙
2'; 'Werner''s P13 A3 Overlay 3'; 'Werner''s P13 A3 Overlay 5'; 'HGPS P5 A1 Overlay↙
2'; 'HGPS P5 A1 Overlay 3'; 'HGPS P5 A1 Overlay 4'; 'HGPS P5 A1 Overlay 5'; 'HGPS P5↙
A2 Overlay 1'; 'HGPS P5 A2 Overlay 2'; 'HGPS P5 A2 Overlay 3'; 'HGPS P5 A2 Overlay 4';↙
'HGPS P5 A2 Overlay 5'; 'HGPS P5 A3 Overlay 1'; 'HGPS P5 A3 Overlay 2'; 'HGPS P5 A3↙
Overlay 3'; 'HGPS P5 A3 Overlay 4'; 'HGPS P5 A3 Overlay 5'; 'HGPS P6 A1 Overlay 1';↙
'HGPS P6 A1 Overlay 2'; 'HGPS P6 A1 Overlay 4'; 'HGPS P6 A1 Overlay 5'; 'HGPS P6 B1↙
Overlay 1'; 'HGPS P6 B1 Overlay 2'; 'HGPS P6 B1 Overlay 3'; 'HGPS P6 B1 Overlay 4';↙
'HGPS P6 B1 Overlay 5'; 'HGPS P6 C1 Overlay 1'; 'HGPS P6 C1 Overlay 2'; 'HGPS P6 C1↙
```

```
Overlay 3'; 'HGPS P6 C1 Overlay 4'; 'HGPS P6 C1 Overlay 5'; 'Cockayne''s P5 A1 Overlay↙
1'; 'Cockayne''s P5 A1 Overlay 2'; 'Cockayne''s P5 A1 Overlay 3'; 'Cockayne''s P5 A1↙
Overlay 4'; 'Cockayne''s P5 A1 Overlay 5'; 'Cockayne''s P5 A2 Overlay 1'; 'Cockayne''s↙
P5 A2 Overlay 2'; 'Cockayne''s P5 A2 Overlay 3'; 'Cockayne''s P5 A2 Overlay 4';↙
'Cockayne''s P5 A2 Overlay 5'; 'Cockayne''s P5 A3 Overlay 1'; 'Cockayne''s P5 A3↙
Overlay 2'; 'Cockayne''s P5 A3 Overlay 3'; 'Cockayne''s P5 A3 Overlay 4'; 'Cockayne''s↙
P5 A3 Overlay 5'; 'Cockayne''s P6 A1 1 Overlay'; 'Cockayne''s P6 A1 2 Overlay';↙
'Cockayne''s P6 A1 3 Overlay'; 'Cockayne''s P6 A1 4 Overlay'; 'Cockayne''s P6 A1 5↙
Overlay'; 'Cockayne''s P6 A2 1 Overlay'; 'Cockayne''s P6 A2 2 Overlay'; 'Cockayne''s↙
P6 A2 3 Overlay'; 'Cockayne''s P6 A2 4 Overlay'; 'Cockayne''s P6 A2 5 Overlay';↙
'Cockayne''s P6 B1 1 Overlay'; 'Cockayne''s P6 B1 2 Overlay'; 'Cockayne''s P6 B1 3↙
Overlay'; 'Cockayne''s P6 B1 4 Overlay'; 'Cockayne''s P6 B1 5 Overlay'; 'Cockayne''s↙
P6 B2 1 Overlay'; 'Cockayne''s P6 B2 2 Overlay'; 'Cockayne''s P6 B2 3 Overlay';↙
'Cockayne''s P6 B2 4 Overlay'; 'Cockayne''s P6 B2 5 Overlay'; 'Cockayne''s P6 C2 1↙
Overlay'; 'Cockayne''s P6 C2 2 Overlay'; 'Cockayne''s P6 C2 3 Overlay'; 'Cockayne''s↙
P6 C2 4 Overlay'; 'Cockayne''s P6 C2 5 Overlay'; 'Cockayne''s P7 A1 Overlay 1';↙
'Cockayne''s P7 A1 Overlay 2'; 'Cockayne''s P7 A1 Overlay 3'; 'Cockayne''s P7 A1↙
Overlay 4'; 'Cockayne''s P7 A1 Overlay 5'; 'Cockayne''s P7 B1 Overlay 1'; 'Cockayne''s↙
P7 B1 Overlay 2'; 'Cockayne''s P7 B1 Overlay 3'; 'Cockayne''s P7 B1 Overlay 4';↙
'Cockayne''s P7 B1 Overlay 5'; 'Cockayne''s P7 C1 Overlay 1'; 'Cockayne''s P7 C1↙
Overlay 2'; 'Cockayne''s P7 C1 Overlay 3'; 'Cockayne''s P7 C1 Overlay 4'; 'Cockayne''s↙
P7 C1 Overlay 5'; 'Cockayne''s P8 A1 Overlay 1'; 'Cockayne''s P8 A1 Overlay 2';↙
'Cockayne''s P8 A1 Overlay 3'; 'Cockayne''s P8 A1 Overlay 4'; 'Cockayne''s P8 A1↙
Overlay 5'; 'Cockayne''s P8 B1 Overlay 1'; 'Cockayne''s P8 B1 Overlay 2'; 'Cockayne''s↙
P8 B1 Overlay 3'; 'Cockayne''s P8 B1 Overlay 4'; 'Cockayne''s P8 B1 Overlay 5';↙
'Cockayne''s P8 C1 Overlay 1'; 'Cockayne''s P8 C1 Overlay 2'; 'Cockayne''s P8 C1↙
Overlay 3'; 'Cockayne''s P8 C1 Overlay 4'; 'Cockayne''s P8 C1 Overlay 5'; 'Cockayne''s↙
P9 A1 Overlay 1'; 'Cockayne''s P9 A1 Overlay 2'; 'Cockayne''s P9 A1 Overlay 3';↙
'Cockayne''s P9 A1 Overlay 4'; 'Cockayne''s P9 A1 Overlay 5'; 'Cockayne''s P9 B1↙
Overlay 1'; 'Cockayne''s P9 B1 Overlay 2'; 'Cockayne''s P9 B1 Overlay 3'; 'Cockayne''s↙
P9 B1 Overlay 4'; 'Cockayne''s P9 B1 Overlay 5'; 'Cockayne''s P9 C1 Overlay 1"';↙
'Cockayne''s P9 C1 Overlay 2"'; 'Cockayne''s P9 C1 Overlay 3"'; 'Cockayne''s P9 C1↙
Overlay 4"'; 'Cockayne''s P9 C1 Overlay 5'; 'Cockayne''s P10 A3 Overlay 1';↙
'Cockayne''s P10 A3 Overlay 2'; 'Cockayne''s P10 A3 Overlay 3'; 'Cockayne''s P10 A3↙
Overlay 4'; 'Cockayne''s P10 A3 Overlay 5'; 'Cockayne''s P10 B1 Overlay 1';↙
'Cockayne''s P10 B1 Overlay 2'; 'Cockayne''s P10 B1 Overlay 3'; 'Cockayne''s P10 B1↙
Overlay 4'; 'Cockayne''s P10 B1 Overlay 5'; 'Cockayne''s P10 C1 Overlay 1';↙
'Cockayne''s P10 C1 Overlay 2'; 'Cockayne''s P10 C1 Overlay 3'; 'Cockayne''s P10 C1↙
Overlay 4'; 'Cockayne''s P10 C1 Overlay 5'; 'Cockayne''s P11 A1 Overlay 1';↙
'Cockayne''s P11 A1 Overlay 2'; 'Cockayne''s P11 A1 Overlay 3'; 'Cockayne''s P11 A1↙
Overlay 4'; 'Cockayne''s P11 A1 Overlay 5'; 'Cockayne''s P11 B1 Overlay 1';↙
'Cockayne''s P11 B1 Overlay 2'; 'Cockayne''s P11 B1 Overlay 3'; 'Cockayne''s P11 B1↙
Overlay 4'; 'Cockayne''s P11 B1 Overlay 5'; 'Cockayne''s P11 C1 Overlay 1';↙
'Cockayne''s P11 C1 Overlay 2'; 'Cockayne''s P11 C1 Overlay 3'; 'Cockayne''s P11 C1↙
Overlay 4'; 'Cockayne''s P11 C1 Overlay 5'; 'Cockayne''s P12 A1 Overlay 1';↙
'Cockayne''s P12 A1 Overlay 2'; 'Cockayne''s P12 A1 Overlay 3'; 'Cockayne''s P12 A1↙
Overlay 4'; 'Cockayne''s P12 A1 Overlay 5'; 'Cockayne''s P12 B1 Overlay 1';↙
'Cockayne''s P12 B1 Overlay 2'; 'Cockayne''s P12 B1 Overlay 3'; 'Cockayne''s P12 B1↙
Overlay 4'; 'Cockayne''s P12 B1 Overlay 5'; 'Cockayne''s P12 C1 Overlay 1';↙
'Cockayne''s P12 C1 Overlay 2'; 'Cockayne''s P12 C1 Overlay 3'; 'Cockayne''s P12 C1↙
Overlay 4'; 'Cockayne''s P12 C1 Overlay 5' },...
'Style','popupmenu',...
'Value',1,...
'ValueMode',get(0,'defaultuicontrolValueMode'),...
'Position',[2 2.4 31.5714285714286 2.2],...
```

```
'BackgroundColor',[1 1 1],...
'Callback',@(hObject,eventdata)v2_0_gui_export('popupmenu_images_Callback',hObject,↙
eventdata,guidata(hObject)),...
'Children',[],...
'CreateFcn', {@local_CreateFcn, @(hObject,eventdata)v2_0_gui_export↙
('popupmenu_images_CreateFcn',hObject,eventdata,guidata(hObject)), appdata} ,...
'Tag','popupmenu_images',...
'FontSize',12);

appdata = [];
appdata.lastValidTag = 'UG_checkbox';

h12 = uicontrol(...
'Parent',h7,...
'Units','characters',...
'FontUnits',get(0,'defaultuicontrolFontUnits'),...
'String','Show H2AX Annotations',...
'Style','checkbox',...
'Position',[46.5714285714286 0.6 21.1428571428571 1.8],...
'Callback',@(hObject,eventdata)v2_0_gui_export('UG_checkbox_Callback',hObject,↙
eventdata,guidata(hObject)),...
'Children',[],...
'ButtonDownFcn',blanks(0),...
'CreateFcn', {@local_CreateFcn, blanks(0), appdata} ,...
'DeleteFcn',blanks(0),...
'Tag','UG_checkbox',...
'KeyPressFcn',blanks(0),...
'FontSize',10,...
'FontSizeMode',get(0,'defaultuicontrolFontSizeMode'));

appdata = [];
appdata.lastValidTag = 'bw_checkbox';

h13 = uicontrol(...
'Parent',h1,...
'Units','characters',...
'FontUnits',get(0,'defaultuicontrolFontUnits'),...
'String','B/W',...
'Style','checkbox',...
'Position',[123.714285714286 52.174358974359 11.8571428571428 2.33333333333334],...
'Callback',@(hObject,eventdata)v2_0_gui_export('bw_checkbox_Callback',hObject,↙
eventdata,guidata(hObject)),...
'Children',[],...
'CreateFcn', {@local_CreateFcn, blanks(0), appdata} ,...
'Tag','bw_checkbox',...
'FontSize',10,...
'FontSizeMode',get(0,'defaultuicontrolFontSizeMode'));

appdata = [];
appdata.lastValidTag = 'ch1_checkbox';

h14 = uicontrol(...
'Parent',h1,...
'Units','characters',...
'FontUnits',get(0,'defaultuicontrolFontUnits'),...
```

```
'String','Ki67 channel',...
'Style','checkbox',...
'Value',1,...
'Position',[123.714285714286 50.0666666666667 14.7142857142857 2.33333333333334],...
'Callback',@(hObject,eventdata)v2_0_gui_export('ch1_checkbox_Callback',hObject,↙
eventdata,guidata(hObject)),...
'Children',[],...
'ButtonDownFcn',blanks(0),...
'CreateFcn', {@local_CreateFcn, blanks(0), appdata} ,...
'DeleteFcn',blanks(0),...
'Tag','ch1_checkbox',...
'KeyPressFcn',blanks(0),...
'FontSize',10,...
'FontSizeMode',get(0,'defaultuicontrolFontSizeMode'));

appdata = [];
appdata.lastValidTag = 'ch2_checkbox';

h15 = uicontrol(...
'Parent',h1,...
'Units','characters',...
'FontUnits',get(0,'defaultuicontrolFontUnits'),...
'String','H2AX channel',...
'Style','checkbox',...
'Value',1,...
'Position',[123.714285714286 47.8666666666667 15 2.33333333333334],...
'Callback',@(hObject,eventdata)v2_0_gui_export('ch2_checkbox_Callback',hObject,↙
eventdata,guidata(hObject)),...
'Children',[],...
'ButtonDownFcn',blanks(0),...
'CreateFcn', {@local_CreateFcn, blanks(0), appdata} ,...
'DeleteFcn',blanks(0),...
'Tag','ch2_checkbox',...
'KeyPressFcn',blanks(0),...
'FontSize',10,...
'FontSizeMode',get(0,'defaultuicontrolFontSizeMode'));

appdata = [];
appdata.lastValidTag = 'ch3_checkbox';

h16 = uicontrol(...
'Parent',h1,...
'Units','characters',...
'FontUnits',get(0,'defaultuicontrolFontUnits'),...
'String','DAPI channel',...
'Style','checkbox',...
'Value',1,...
'Position',[123.714285714286 45.5333333333333 14.4285714285714 2.33333333333334],...
'Callback',@(hObject,eventdata)v2_0_gui_export('ch3_checkbox_Callback',hObject,↙
eventdata,guidata(hObject)),...
'Children',[],...
'ButtonDownFcn',blanks(0),...
'CreateFcn', {@local_CreateFcn, blanks(0), appdata} ,...
'DeleteFcn',blanks(0),...
'Tag','ch3_checkbox',...
```

```
'KeyPressFcn',blanks(0),...
'FontSize',10,...
'FontSizeMode',get(0,'defaultuicontrolFontSizeMode'));

appdata = [];
appdata.lastValidTag = 'uitoolbar1';

h17 = uitoolbar(...
'Parent',h1,...
'Tag','uitoolbar1',...
'CreateFcn', {@local_CreateFcn, blanks(0), appdata} );

appdata = [];
appdata.toolid = 'Exploration.ZoomIn';
appdata.CallbackInUse = struct(...
    'ClickedCallback', '%default');
appdata.lastValidTag = 'uitoggletool1';

h18 = uitoggletool(...
'Parent',h17,...
'Serializable',get(0,'defaultuitoggletoolSerializable'),...
'Children',[],...
'HandleVisibility',get(0,'defaultuitoggletoolHandleVisibility'),...
'Tag','uitoggletool1',...
'Tooltip','Zoom In',...
'TooltipMode',get(0,'defaultuitoggletoolTooltipMode'),...
'CData',mat{1},...
'ClickedCallback','%default',...
'TooltipString','Zoom In',...
'CreateFcn', {@local_CreateFcn, blanks(0), appdata} );

appdata = [];
appdata.toolid = 'Exploration.ZoomOut';
appdata.CallbackInUse = struct(...
    'ClickedCallback', '%default');
appdata.lastValidTag = 'uitoggletool2';

h19 = uitoggletool(...
'Parent',h17,...
'Serializable',get(0,'defaultuitoggletoolSerializable'),...
'Children',[],...
'HandleVisibility',get(0,'defaultuitoggletoolHandleVisibility'),...
'Tag','uitoggletool2',...
'Tooltip','Zoom Out',...
'TooltipMode',get(0,'defaultuitoggletoolTooltipMode'),...
'CData',mat{2},...
'ClickedCallback','%default',...
'TooltipString','Zoom Out',...
'CreateFcn', {@local_CreateFcn, blanks(0), appdata} );

appdata = [];
appdata.toolid = 'Exploration.Pan';
appdata.CallbackInUse = struct(...
    'ClickedCallback', '%default');
appdata.lastValidTag = 'uitoggletool3';
```

```
h20 = uitoggletool(...
'Parent',h17,...
'Serializable',get(0,'defaultuitoggletoolSerializable'),...
'Children',[],...
'HandleVisibility',get(0,'defaultuitoggletoolHandleVisibility'),...
'Tag','uitoggletool3',...
'Tooltip','Pan',...
'TooltipMode',get(0,'defaultuitoggletoolTooltipMode'),...
'CData',mat{3},...
'ClickedCallback','%default',...
'TooltipString','Pan',...
'CreateFcn', {@local_CreateFcn, blanks(0), appdata} );

appdata = [];
appdata.toolid = 'Exploration.DataCursor';
appdata.CallbackInUse = struct(...
    'ClickedCallback', '%default');
appdata.lastValidTag = 'uitoggletool4';

h21 = uitoggletool(...
'Parent',h17,...
'Serializable',get(0,'defaultuitoggletoolSerializable'),...
'Children',[],...
'HandleVisibility',get(0,'defaultuitoggletoolHandleVisibility'),...
'Tag','uitoggletool4',...
'Tooltip','Data Cursor',...
'TooltipMode',get(0,'defaultuitoggletoolTooltipMode'),...
'CData',mat{4},...
'ClickedCallback','%default',...
'TooltipString','Data Cursor',...
'CreateFcn', {@local_CreateFcn, blanks(0), appdata} );


hsingleton = h1;


% --- Set application data first then calling the CreateFcn.
function local_CreateFcn(hObject, eventdata, createfcn, appdata)

if ~isempty(appdata)
   names = fieldnames(appdata);
   for i=1:length(names)
       name = char(names(i));
       setappdata(hObject, name, getfield(appdata,name));
   end
end

if ~isempty(createfcn)
   if isa(createfcn,'function_handle')
       createfcn(hObject, eventdata);
   else
       eval(createfcn);
   end
end
```

```
% --- Handles default GUIDE GUI creation and callback dispatch
function varargout = gui_mainfcn(gui_State, varargin)

gui_StateFields =  {'gui_Name'
    'gui_Singleton'
    'gui_OpeningFcn'
    'gui_OutputFcn'
    'gui_LayoutFcn'
    'gui_Callback'};
gui_Mfile = '';
for i=1:length(gui_StateFields)
    if ~isfield(gui_State, gui_StateFields{i})
        error(message('MATLAB:guide:StateFieldNotFound', gui_StateFields{ i },↙
gui_Mfile));
    elseif isequal(gui_StateFields{i}, 'gui_Name')
        gui_Mfile = [gui_State.(gui_StateFields{i}), '.m'];
    end
end

numargin = length(varargin);

if numargin == 0
    % V2_0_GUI_EXPORT
    % create the GUI only if we are not in the process of loading it
    % already
    gui_Create = true;
elseif local_isInvokeActiveXCallback(gui_State, varargin{:})
    % V2_0_GUI_EXPORT(ACTIVEX,...)
    vin{1} = gui_State.gui_Name;
    vin{2} = [get(varargin{1}.Peer, 'Tag'), '_', varargin{end}];
    vin{3} = varargin{1};
    vin{4} = varargin{end-1};
    vin{5} = guidata(varargin{1}.Peer);
    feval(vin{:});
    return;
elseif local_isInvokeHGCallback(gui_State, varargin{:})
    % V2_0_GUI_EXPORT('CALLBACK',hObject,eventData,handles,...)
    gui_Create = false;
else
    % V2_0_GUI_EXPORT(...)
    % create the GUI and hand varargin to the openingfcn
    gui_Create = true;
end

if ~gui_Create
    % In design time, we need to mark all components possibly created in
    % the coming callback evaluation as non-serializable. This way, they
    % will not be brought into GUIDE and not be saved in the figure file
    % when running/saving the GUI from GUIDE.
    designEval = false;
    if (numargin>1 && ishghandle(varargin{2}))
        fig = varargin{2};
        while ~isempty(fig) && ~ishghandle(fig,'figure')
```

```
            fig = get(fig,'parent');
        end

        designEval = isappdata(0,'CreatingGUIDEFigure') || (isscalar(fig)&&isprop↙
(fig,'GUIDEFigure'));
    end

    if designEval
        beforeChildren = findall(fig);
    end

    % evaluate the callback now
    varargin{1} = gui_State.gui_Callback;
    if nargout
        [varargout{1:nargout}] = feval(varargin{:});
    else
        feval(varargin{:});
    end

    % Set serializable of objects created in the above callback to off in
    % design time. Need to check whether figure handle is still valid in
    % case the figure is deleted during the callback dispatching.
    if designEval && ishghandle(fig)
        set(setdiff(findall(fig),beforeChildren), 'Serializable','off');
    end
else
    if gui_State.gui_Singleton
        gui_SingletonOpt = 'reuse';
    else
        gui_SingletonOpt = 'new';
    end

    % Check user passing 'visible' P/V pair first so that its value can be
    % used by oepnfig to prevent flickering
    gui_Visible = 'auto';
    gui_VisibleInput = '';
    for index=1:2:length(varargin)
        if length(varargin) == index || ~ischar(varargin{index})
            break;
        end

        % Recognize 'visible' P/V pair
        len1 = min(length('visible'),length(varargin{index}));
        len2 = min(length('off'),length(varargin{index+1}));
        if ischar(varargin{index+1}) && strncmpi(varargin{index},'visible',len1) &&↙
len2 > 1
            if strncmpi(varargin{index+1},'off',len2)
                gui_Visible = 'invisible';
                gui_VisibleInput = 'off';
            elseif strncmpi(varargin{index+1},'on',len2)
                gui_Visible = 'visible';
                gui_VisibleInput = 'on';
            end
        end
    end
```

```
    % Open fig file with stored settings.  Note: This executes all component
    % specific CreateFunctions with an empty HANDLES structure.


    % Do feval on layout code in m-file if it exists
    gui_Exported = ~isempty(gui_State.gui_LayoutFcn);
    % this application data is used to indicate the running mode of a GUIDE
    % GUI to distinguish it from the design mode of the GUI in GUIDE. it is
    % only used by actxproxy at this time.
    setappdata(0,genvarname(['OpenGuiWhenRunning_', gui_State.gui_Name]),1);
    if gui_Exported
        gui_hFigure = feval(gui_State.gui_LayoutFcn, gui_SingletonOpt);

        % make figure invisible here so that the visibility of figure is
        % consistent in OpeningFcn in the exported GUI case
        if isempty(gui_VisibleInput)
            gui_VisibleInput = get(gui_hFigure,'Visible');
        end
        set(gui_hFigure,'Visible','off')

        % openfig (called by local_openfig below) does this for guis without
        % the LayoutFcn. Be sure to do it here so guis show up on screen.
        movegui(gui_hFigure,'onscreen');
    else
        gui_hFigure = local_openfig(gui_State.gui_Name, gui_SingletonOpt,↙
gui_Visible);
        % If the figure has InGUIInitialization it was not completely created
        % on the last pass.  Delete this handle and try again.
        if isappdata(gui_hFigure, 'InGUIInitialization')
            delete(gui_hFigure);
            gui_hFigure = local_openfig(gui_State.gui_Name, gui_SingletonOpt,↙
gui_Visible);
        end
    end
    if isappdata(0, genvarname(['OpenGuiWhenRunning_', gui_State.gui_Name]))
        rmappdata(0,genvarname(['OpenGuiWhenRunning_', gui_State.gui_Name]));
    end

    % Set flag to indicate starting GUI initialization
    setappdata(gui_hFigure,'InGUIInitialization',1);

    % Fetch GUIDE Application options
    gui_Options = getappdata(gui_hFigure,'GUIDEOptions');
    % Singleton setting in the GUI MATLAB code file takes priority if different
    gui_Options.singleton = gui_State.gui_Singleton;

    if ~isappdata(gui_hFigure,'GUIOnScreen')
        % Adjust background color
        if gui_Options.syscolorfig
            set(gui_hFigure,'Color', get(0,'DefaultUicontrolBackgroundColor'));
        end

        % Generate HANDLES structure and store with GUIDATA. If there is
        % user set GUI data already, keep that also.
```

```
        data = guidata(gui_hFigure);
        handles = guihandles(gui_hFigure);
        if ~isempty(handles)
            if isempty(data)
                data = handles;
            else
                names = fieldnames(handles);
                for k=1:length(names)
                    data.(char(names(k)))=handles.(char(names(k)));
                end
            end
        end
        guidata(gui_hFigure, data);
    end

    % Apply input P/V pairs other than 'visible'
    for index=1:2:length(varargin)
        if length(varargin) == index || ~ischar(varargin{index})
            break;
        end

        len1 = min(length('visible'),length(varargin{index}));
        if ~strncmpi(varargin{index},'visible',len1)
            try set(gui_hFigure, varargin{index}, varargin{index+1}), catch break, end
        end
    end

    % If handle visibility is set to 'callback', turn it on until finished
    % with OpeningFcn
    gui_HandleVisibility = get(gui_hFigure,'HandleVisibility');
    if strcmp(gui_HandleVisibility, 'callback')
        set(gui_hFigure,'HandleVisibility', 'on');
    end

    feval(gui_State.gui_OpeningFcn, gui_hFigure, [], guidata(gui_hFigure), varargin↙
{:});

    if isscalar(gui_hFigure) && ishghandle(gui_hFigure)
        % Handle the default callbacks of predefined toolbar tools in this
        % GUI, if any
        guidemfile('restoreToolbarToolPredefinedCallback',gui_hFigure);

        % Update handle visibility
        set(gui_hFigure,'HandleVisibility', gui_HandleVisibility);

        % Call openfig again to pick up the saved visibility or apply the
        % one passed in from the P/V pairs
        if ~gui_Exported
            gui_hFigure = local_openfig(gui_State.gui_Name, 'reuse',gui_Visible);
        elseif ~isempty(gui_VisibleInput)
            set(gui_hFigure,'Visible',gui_VisibleInput);
        end
        if strcmpi(get(gui_hFigure, 'Visible'), 'on')
            figure(gui_hFigure);
```

```
            if gui_Options.singleton
                setappdata(gui_hFigure,'GUIOnScreen', 1);
            end
        end

        % Done with GUI initialization
        if isappdata(gui_hFigure,'InGUIInitialization')
            rmappdata(gui_hFigure,'InGUIInitialization');
        end

        % If handle visibility is set to 'callback', turn it on until
        % finished with OutputFcn
        gui_HandleVisibility = get(gui_hFigure,'HandleVisibility');
        if strcmp(gui_HandleVisibility, 'callback')
            set(gui_hFigure,'HandleVisibility', 'on');
        end
        gui_Handles = guidata(gui_hFigure);
    else
        gui_Handles = [];
    end

    if nargout
        [varargout{1:nargout}] = feval(gui_State.gui_OutputFcn, gui_hFigure, [],↙
gui_Handles);
    else
        feval(gui_State.gui_OutputFcn, gui_hFigure, [], gui_Handles);
    end

    if isscalar(gui_hFigure) && ishghandle(gui_hFigure)
        set(gui_hFigure,'HandleVisibility', gui_HandleVisibility);
    end
end

function gui_hFigure = local_openfig(name, singleton, visible)

% openfig with three arguments was new from R13. Try to call that first, if
% failed, try the old openfig.
if nargin('openfig') == 2
    % OPENFIG did not accept 3rd input argument until R13,
    % toggle default figure visible to prevent the figure
    % from showing up too soon.
    gui_OldDefaultVisible = get(0,'defaultFigureVisible');
    set(0,'defaultFigureVisible','off');
    gui_hFigure = matlab.hg.internal.openfigLegacy(name, singleton);
    set(0,'defaultFigureVisible',gui_OldDefaultVisible);
else
    % Call version of openfig that accepts 'auto' option"
    gui_hFigure = matlab.hg.internal.openfigLegacy(name, singleton, visible);
%      %workaround for CreateFcn not called to create ActiveX
%          peers=findobj(findall(allchild↙
(gui_hFigure)),'type','uicontrol','style','text');
%          for i=1:length(peers)
%              if isappdata(peers(i),'Control')
%                  actxproxy(peers(i));
%              end
```

```
%           end
end

function result = local_isInvokeActiveXCallback(gui_State, varargin)

try
    result = ispc && iscom(varargin{1}) ...
             && isequal(varargin{1},gcbo);
catch
    result = false;
end

function result = local_isInvokeHGCallback(gui_State, varargin)

try
    fhandle = functions(gui_State.gui_Callback);
    result = ~isempty(findstr(gui_State.gui_Name,fhandle.file)) || ...
             (ischar(varargin{1}) ...
             && isequal(ishghandle(varargin{2}), 1) ...
             && (~isempty(strfind(varargin{1},[get(varargin{2}, 'Tag'), '_'])) || ...
                ~isempty(strfind(varargin{1}, '_CreateFcn'))) );
catch
    result = false;
end
```

```
function [imgout]=JS_loadLif2(filename)
% modiified version ... ci_loadLif - Load Leica Image File Format
%
% Based on HKLoadlif by Hiroshi Kawaguchi
% http://de.mathworks.com/matlabcentral/fileexchange/36005-leica-image-format-file-↙
loader
%
% [imgout]=ci_loadLif('filename', getonlynumberofelements, number) loads an image↙
(series) from a Leica Image File
% imgout =
%                Image: {[1024x1024x101 uint8]}
%                 Info: [1x1 struct]
%                 Name: 'loc1'
%                 Type: 'X-Y-Z'
%     NumberOfChannel: 1
%                 Size: '1024  1024   101'
%
% diplibtensor=dip_image(imgout.Image); gives dipimage Tensor Image
% diplibimage=array2im(diplibtensor); gives nCh image
% channelimage=diplibimage(:,:,:,ch); gives ch image

%
% History
% Version 1.0
% Some LAS-AF 4.x files give errors, fixed in LAS-X

% (c) Ron Hoebe
% Cellular Imaging - Core Facility
% AMC - UvA - Amsterdam - The Netherlands

%% Main function

if nargin==0
    [filename, pathname]=uigetfile({'*.lif','Leica Image Format (*.lif)'});
    if filename==0; return; end
    filename=[pathname filename];
%     getonlynumberofelements=false;
%     number=1;
end
fp=fopen(filename,'r');

% Reading XML Part
[fp, xmlHdrStr] = ReadXMLPart(fp);

% xmlList is cell array (n x 5)
% rank(double) name(string) attributes(cell(n,2)) parant(double) children(double↙
array)
% Changing XML to Cell
xmlList=XMLtxt2cell(xmlHdrStr);
lifVersion = GetLifVersion(xmlList(1,:)); % lifVersion is double scalar

% Reading Image Info
imgList = GetImageDescriptionList(xmlList);% imgList is struct vector
% memoryList is cell array (n x 4)
% ID(string), startPoint(uint64), sizeOfMemory, Index(double)
```

```
imgList  = ReadObjectMemoryBlocks(fp,lifVersion,imgList);
fclose(fp);

dat=cell(numel(imgList),5);
for n=1:numel(imgList)
    dat{n,1}=imgList(n).Name;            % Name of image
    dat{n,2}=numel(imgList(n).Channels);% number of channel
    [dimType, dimSize]=GetDimensionInfo(imgList(n).Dimensions);
    dat{n,3}=dimType;
    dat{n,4}=int2str(dimSize');
    dat{n,5}=false;
end

j = 1;
for i = 1:numel(imgList)
str = imgList(i).Name;
pat1 = "Overlay"; pat2 = "overlay"; TF1 = contains(str,pat1); TF2 = contains(str,↙
pat2);
if TF1 == 1
  LS{j,1} = str;
  IM  = ReadAnImageData(imgList(i), filename);
  IS = ReconstructImage(imgList(i), IM);
  LS{j,2} = IS.Image{1, 1}; LS{j,3} = IS.Image{2, 1}; LS{j,4} = IS.Image{3, 1};
  j = j+1;
elseif TF2 == 1
  LS{j,1} = str;
  IM  = ReadAnImageData(imgList(i), filename);
  IS = ReconstructImage(imgList(i), IM);
  LS{j,2} = IS.Image{1, 1}; LS{j,3} = IS.Image{2, 1}; LS{j,4} = IS.Image{3, 1};
  j = j+1;
end
end
imgout = LS;

return


function [dimType, dimSize]=GetDimensionInfo(dimensions)
ndims=numel(dimensions);
dimType=cell(2*ndims,1);
dimSize=zeros(ndims,1);

for n=1:ndims
    dimSize(n)=str2double(dimensions(n).NumberOfElements);
    switch dimensions(n).DimID
        case '0';dimType{2*n-1}='Not Valied';
        case '1';dimType{2*n-1}='X';
        case '2';dimType{2*n-1}='Y';
        case '3';dimType{2*n-1}='Z';
        case '4';dimType{2*n-1}='T';
        case '5';dimType{2*n-1}='Lambda';
        case '6';dimType{2*n-1}='Rotation';
        case '7';dimType{2*n-1}='XT';
        case '8';dimType{2*n-1}='TSlice';
        otherwise
```

```
    end
    dimType{2*n}='-';
end
dimType=strrep(sprintf('%s',char(dimType)'),' ','');
dimType=dimType(1:end-1);% Delete last '-'

function imgList=ReconstructImage(imgInfo, imgData)
% ==================================================================
% imgInfo description
% ==================================================================
% <ChannelDescription>
%%% DataType   [0, 1]                [Integer, Float]
%%% ChannelTag [0, 1, 2, 3]          [GrayValue, Red, Green, Blue]
% Resolution [Unsigned integer]   Bits per pixel if DataType is Float value can be 32↙
or 64 (float or double)
% NameOfMeasuredQuantity [String] Name
% Min        [Double] Physical Value of the lowest gray value (0). If DataType is↙
Float the Minimal possible value (or 0).
% Max        [Double] Physical Value of the highest gray value (e.g. 255) If DataType↙
is Float the Maximal possible value (or 0).
% Unit       [String] Physical Unit
% LUTName    [String] Name of the Look Up Table (Gray value to RGB value)
% IsLUTInverted [0, 1] Normal LUT Inverted Order
%%% BytesInc   [Unsigned long (64 Bit)] Distance from the first channel in Bytes
% BitInc     [Unsigned Integer]      Bit Distance for some RGB Formats (not used in↙
LAS AF 1..0 ? 1.7)
% <DimensionDescription>
% DimID   [0, 1, 2, 3, 4, 5, 6, 7, 8] [Not valid, X, Y, Z, T, Lambda, Rotation, XT↙
Slices, T Slices]
%%% NumberOfElements [Unsigned Integer] Number of elements in this dimension
% Origin           [Unsigned integer] Physical position of the first element (Left↙
pixel side)
% Length    [String] Physical Length from the first left pixel side to the last left↙
pixel side (Not the right. A Pixel has no width!)
% Unit      [String] Physical Unit
%%% BytesInc [Unsigned long (64 Bit)] Distance from one Element to the next in this↙
dimension
% BitInc    [Unsigned Integer] Bit Distance for some RGB Formats (not used, i.e.: = 0↙
in LAS AF 1..0 ? 1.7)
% ==================================================================
% imgList info
% ==================================================================
% img

% Get Dimension info
dimension=ones(1,9);
for m=1:numel(imgInfo.Dimensions)
    dimension(str2double(imgInfo.Dimensions(m).DimID))=str2double(imgInfo.Dimensions↙
(m).NumberOfElements);
end

% Separate to each channel image
nCh=numel(imgInfo.Channels);
imgList=struct('Image',[],'Info',[]);
imgList.Image = cell(nCh,1);
```

```
if nCh > 1;
    imgData=reshape(imgData,...
        str2double(imgInfo.Channels(2).BytesInc)-str2double(imgInfo.Channels(1).↙
BytesInc),[]);
    for m=1:nCh
        tmp=imgData(:,m:nCh:end);
        imgList.Image{m}= reshape(typecast(tmp(:),GetType(imgInfo.Channels(m))),↙
dimension);
    end
else
    imgList.Image{1}=reshape(typecast(imgData,GetType(imgInfo.Channels)),dimension);
end
imgList.Info = imgInfo;

function chType=GetType(Channels)
switch str2double(Channels.DataType)
    case 0 % int case
        switch str2double(Channels.Resolution);
            % currently, resolution is constant through the channels
            case 8;   chType='uint8';
            case 12;  chType='uint16';
            case 32;  chType='uint32';
            case 64;  chType='uint64';
            otherwise;error('Unsupported data bit. ')
        end
    case 1 % float case
        switch str2double(Channels.Resolution);
            % currently, resolution is constant through the channels
            case 32;  chType='single';
            case 64;  chType='double';
            otherwise;error('Unsupported data bit. ')
        end
end

function mems = GetImageDescriptionList(xmlList)
%  For the image data type the description of the memory layout is defined
%  in the image description XML node (<ImageDescription>).

% <ImageDescription>
imgIndex  =SearchTag(xmlList,'ImageDescription');
numImgs=numel(imgIndex);

% <Memory Size="21495808" MemoryBlockID="MemBlock_233"/>
memIndex  =SearchTag(xmlList,'Memory');
memSizes  =cellfun(@str2double,GetAttributeVal(xmlList,memIndex,'Size'));
memIndex=memIndex(memSizes~=0);
memSizes=memSizes(memSizes~=0);
if numImgs~=numel(memIndex);
    error('Number of ImageDescription and Memory did not match.')
end

% Matching ImageDescription with Memory
imgParentElmIndex =  zeros(numImgs,1);
for n=1:numImgs
    imgParentElmIndex(n)  = SearchTagParent(xmlList,imgIndex(n),'Element');
```

```
end
memParentElmIndex =  zeros(numImgs,1);
for n=1:numImgs
    memParentElmIndex(n) = SearchTagParent(xmlList,memIndex(n),'Element');
end
[imgParentElmIndex, sortIndex]=sort(imgParentElmIndex); imgIndex=imgIndex(sortIndex);
[memParentElmIndex, sortIndex]=sort(memParentElmIndex); memIndex=memIndex(sortIndex);↙
memSizes=memSizes(sortIndex);
if ~all(imgParentElmIndex==memParentElmIndex)
    error('Matching ImageDescriptions with Memories')
end

mems=struct('Name',[],'Channels',[],'Dimensions',[],'Memory',[]);
mems(numImgs).Memory.StartPosition=[];
for n=1:numImgs
    mems(n).Name = char(GetAttributeVal(xmlList, imgParentElmIndex(n),'Name'));
    [mems(n).Channels, mems(n).Dimensions]= MakeImageStruct(xmlList,imgIndex(n));
    mems(n).Memory.Size=memSizes(n);
    mems(n).Memory.MemoryBlockID=char(GetAttributeVal(xmlList,memIndex↙
(n),'MemoryBlockID'));
end


return

function [C, D]=MakeImageStruct(xmlList,iid)
% ChannelDescription   DataType="0" ChannelTag="0" Resolution="8"
%                      NameOfMeasuredQuantity="" Min="0.000000e+000" Max="2.550000↙
e+002"
%                      Unit="" LUTName="Red" IsLUTInverted="0" BytesInc="0"
%                      BitInc="0"
% DimensionDescription DimID="1" NumberOfElements="512" Origin="4.336809e-020"
%                      Length="4.558820e-004" Unit="m" BitInc="0"
%                      BytesInc="1"
% Memory ?@?@?@?@?@?@?@  Size="21495808" MemoryBlockID="MemBlock_233"
iidChildren=xmlList{iid,5};
for n=1:numel(iidChildren)
    if strcmp(xmlList{iidChildren(n),2},'Channels')
        id=xmlList{iidChildren(n),5};
        p=xmlList(id,3);
        nid=numel(id);
        tmp=cell(11,nid);
        for m=1:nid
            tmp(:,m)=p{m}(:,2);
        end
        C=cell2struct(tmp,p{1}(:,1),1);
    elseif strcmp(xmlList{iidChildren(n),2},'Dimensions')
        id=xmlList{iidChildren(n),5};
        p=xmlList(id,3);
        nid=numel(id);
        tmp=cell(7,nid);
        for m=1:nid
            tmp(:,m)=p{m}(:,2);
        end
        D=cell2struct(tmp,p{1}(:,1),1);
```

```
    else
        error('Undefined Tag')
    end
end

function lifVersion = GetLifVersion(xmlList)
% return version of header
index  =SearchTag(xmlList,'LMSDataContainerHeader');
value  =GetAttributeVal(xmlList,index,'Version');
lifVersion = str2double(cell2mat(value(1)));
return

function pindex=SearchTagParent(xmlList,index,tagName)
% return the row index of given tag name
pindex=xmlList{index,4};

while pindex~=0
    if strcmp(xmlList{pindex,2},tagName)
        return;
    else
        pindex=xmlList{pindex,4};
    end
end
error('Cannot Find the Parent Tag "%s"',tagName);
return;

function index=SearchTag(xmlList,tagName)
% return the row index of given tag name
listLen=size(xmlList,1);
index=[];
for n=1:listLen
    if strcmp(char(xmlList(n,2)),tagName)
        index=[index; n]; %#ok<AGROW>
    end
end

function value=GetAttributeVal(xmlList, index, attributeName)
% return cell array of attributes row index of given tag name
value={};
for n=1:length(index)
    currentCell=xmlList{index(n),3};
    for m=1:size(currentCell,1)
        if strcmp(char(currentCell(m,1)),attributeName)
            value=[value; currentCell(m,2)]; %#ok<AGROW>
        end
    end
end

function CheckTestValue(value,errorMsg)
switch class(value)
    case 'uint8';  trueVal=hex2dec('2A');
    case 'uint32'; trueVal=hex2dec('70');
    otherwise;
        error('Unsupported Error Number: %d',value)
end
```

```
if value~=trueVal
    error(errorMsg);
end
return;

function [fp, str, ketPos] = ReadXMLPart(fp)
% Size(bytes) Total(bytes) description (some LAS-AF 3.x version give errors
% here, fixed in LAS-X
CheckTestValue(fread(fp,1,'*uint32'),...          % 4   4 Test Value 0x70
    'Invalid test value at Part: XML.');
xmlChunk = fread(fp, 1, 'uint32');                % 4   8 Binary Chunk length NC*2 + 1 +↙
4
CheckTestValue(fread(fp,1,'*uint8'),...           % 1   9 Test Value 0x2A
    'Invalid test value at XML Content.');
nc = fread(fp,1,'uint32');                        % 4 13 Number of UTF-16 Characters↙
(NC)
if (nc*2 + 1 + 4)~=xmlChunk;  %
    error('Chunk size mismatch at Part: XML.');
end
str= fread(fp,nc*2,'char');                       % 2*nc - XML Object Description

% UTF-16 -> UTF-8 (cut zeros)
str     = char(str(1:2:end)');
% Insert linefeed(char(10)) for facilitate visualization -----
% str=strrep(str,'><',['>' char(10) '<']);
ketPos =strfind(str,'>'); % find position of ">" for fast search of element
return;

function imgLists=ReadObjectMemoryBlocks(fp,lifVersion,imgLists)
% get end of file and return current point
cofp=    ftell(fp);
fseek(fp,0,'eof');
eofp=    ftell(fp);
fseek(fp,cofp,'bof');

nImgLists=length(imgLists);
memoryList=cell(nImgLists,4);
% ID(string), startPoint(uint64), sizeOfMemory, Index(double)
for n = 1:nImgLists
    memoryList{n,1}=imgLists(n).Memory.MemoryBlockID;
end

% read object memory blocks
while ftell(fp) < eofp;

    CheckTestValue(fread(fp,1,'*uint32'),...          % Test Value 0x70
        'Invalied test value at Object Memory Block');

    objMemBlkChunk = fread(fp, 1, '*uint32');%#ok<NASGU> % Size of Description

    CheckTestValue(fread(fp,1,'*uint8'),...           % Test Value 0x2A
        'Invalied test value at Object Memory Block');


    switch uint8(lifVersion)            % Size of Memory (version dependent)
```

```
        case 1; sizeOfMemory = double(fread(fp, 1, '*uint32'));
        case 2; sizeOfMemory = double(fread(fp, 1, '*uint64'));
        otherwise; error('Unsupported LIF version. Update this program');
    end

    CheckTestValue(fread(fp,1,'*uint8'),...          % Test Value 0x2A
        'Invalied test value at Object Memory Block');

    nc = fread(fp,1,'*uint32');                      % Number of MemoryID string

    str = fread(fp,nc*2,'*char')';                   % Number of MemoryID string (UTF-↙
16)
    str = char(str(1:2:end));                        % convert UTF-16 to UTF-8

    if sizeOfMemory > 0
        for n=1:nImgLists
            if strcmp(char(memoryList{n,1}),str) % NEED CONSIDERATION !!!!!!
                if imgLists(n).Memory.Size ~= sizeOfMemory;
                    error('Memory Size Mismatch.');
                end
                imgLists(n).Memory.StartPosition=ftell(fp);
                fseek(fp,sizeOfMemory,'cof');
                break;
            end
        end
    end
end


return;

function imgData=ReadAnImageData(imgInfo,fileName)
fp = fopen(fileName,'rb');
if fp<0; errordlg('Cannot open file: \n\t%s', fileName); end
fseek(fp,imgInfo.Memory.StartPosition,'bof');
imgData = fread(fp,imgInfo.Memory.Size,'*uint8');
fclose(fp);

function tagList=XMLtxt2cell(c)
% rank(double) name(string) attributes(cell(n,2)) parant(double) children(double↙
array)
tags  =regexp(c,'<("[^"]*"|''[^'']*''|[^''>])*>','match')';
nTags=numel(tags);
tagList=cell(nTags,5);
tagRank=0;
tagCount=0;
for n=1:nTags
    currentTag=tags{n}(2:end-1);
    if currentTag(1)=='/'
        tagRank=tagRank-1;
        continue;
    end
    tagRank=tagRank+1;
    tagCount=tagCount+1;
    [tagName, attributes]=ParseTagString(currentTag);
```

```
    tagList{tagCount,1}=tagRank;
    tagList{tagCount,2}=tagName;
    tagList{tagCount,3}=attributes;
    % search parant
    if tagRank~=1
        if tagRank~=tagList{tagCount-1,1};
            tagRankList=cell2mat(tagList(1:tagCount,1));
            parent=find(tagRankList==tagRank-1,1,'last');
            tagList{tagCount,4}=parent;
        else
            tagList{tagCount,4}=tagList{tagCount-1,4};
        end
    else
        tagList{tagCount,4} = 0;
    end
    if currentTag(end)=='/'
        tagRank=tagRank-1;
    end
end

tagList   =tagList(1:tagCount,:);
parentList=cell2mat(tagList(:,4));
% Make Children List
for n=1:tagCount
    tagList{n,5}=find(parentList==n);
end

return;

function [name, attributes]=ParseTagString(tag)
[name, tmpAttributes]=regexp(tag,'^\w+','match', 'split');
name=char(name);
attributesCell=regexp(char(tmpAttributes(end)),'\w+=".*?"','match');
if isempty(attributesCell)
    attributes={};
else
    nAttributes = numel(attributesCell);
    attributes=cell(nAttributes,2);
    for n=1:nAttributes
        currAttrib=char(attributesCell(n));
        dqpos=strfind(currAttrib,'"');
        attributes{n,1}=currAttrib(1:dqpos(1)-2);
        if dqpos(2)-dqpos(1)==1 % case attribute=""
            attributes{n,2}='';
        else
            attributes{n,2}=currAttrib(dqpos(1)+1:dqpos(2)-1);
        end
    end
end
```
